# Control of serine integrase recombination directionality by fusion with the directionality factor

**DOI:** 10.1093/nar/gkx567

**Published:** 2017-06-28

**Authors:** Femi J. Olorunniji, Arlene L. McPherson, Susan J. Rosser, Margaret C.M. Smith, Sean D. Colloms, W. Marshall Stark

**Affiliations:** 1 Institute of Molecular, Cell and Systems Biology, University of Glasgow, Bower Building, Glasgow G12 8QQ, UK; 2 SynthSys - Synthetic and Systems Biology, School of Biological Sciences, University of Edinburgh, Roger Land Building, The King's Buildings, Mayfield Road, Edinburgh EH9 3JD, UK; 3 Department of Biology, University of York, Wentworth Way, York YO10 5DD, UK

## Abstract

Bacteriophage serine integrases are extensively used in biotechnology and synthetic biology for assembly and rearrangement of DNA sequences. Serine integrases promote recombination between two different DNA sites, *attP* and *attB*, to form recombinant *attL* and *attR* sites. The ‘reverse’ reaction requires another phage-encoded protein called the recombination directionality factor (RDF) in addition to integrase; RDF activates *attL* × *attR* recombination and inhibits *attP* × *attB* recombination. We show here that serine integrases can be fused to their cognate RDFs to create single proteins that catalyse efficient *attL* × *attR* recombination *in vivo* and *in vitro*, whereas *attP* × *attB* recombination efficiency is reduced. We provide evidence that activation of *attL* × *attR* recombination involves intra-subunit contacts between the integrase and RDF moieties of the fusion protein. Minor changes in the length and sequence of the integrase–RDF linker peptide did not affect fusion protein recombination activity. The efficiency and single-protein convenience of integrase–RDF fusion proteins make them potentially very advantageous for biotechnology/synthetic biology applications. Here, we demonstrate efficient gene cassette replacement in a synthetic metabolic pathway gene array as a proof of principle.

## INTRODUCTION

The rapidly advancing fields of biotechnology and synthetic biology demand ever more sophisticated and efficient tools for precise manipulation of DNA. Often, double-stranded DNA molecules must be cut at defined positions and rejoined in new ways, for example to assemble long arrays of genes from shorter DNA molecules, integrate specific transgene sequences into precise genomic locations, or rearrange regulatory sequences to alter gene expression. The natural ‘DNA cut and paste’ enzymes known as site-specific recombinases (SSRs) have been much exploited for these applications (1). Recently the serine integrases, a family of SSRs derived from bacteriophages (2), have been used in many applications because of their uniquely advantageous directional properties.

**Figure 1.**
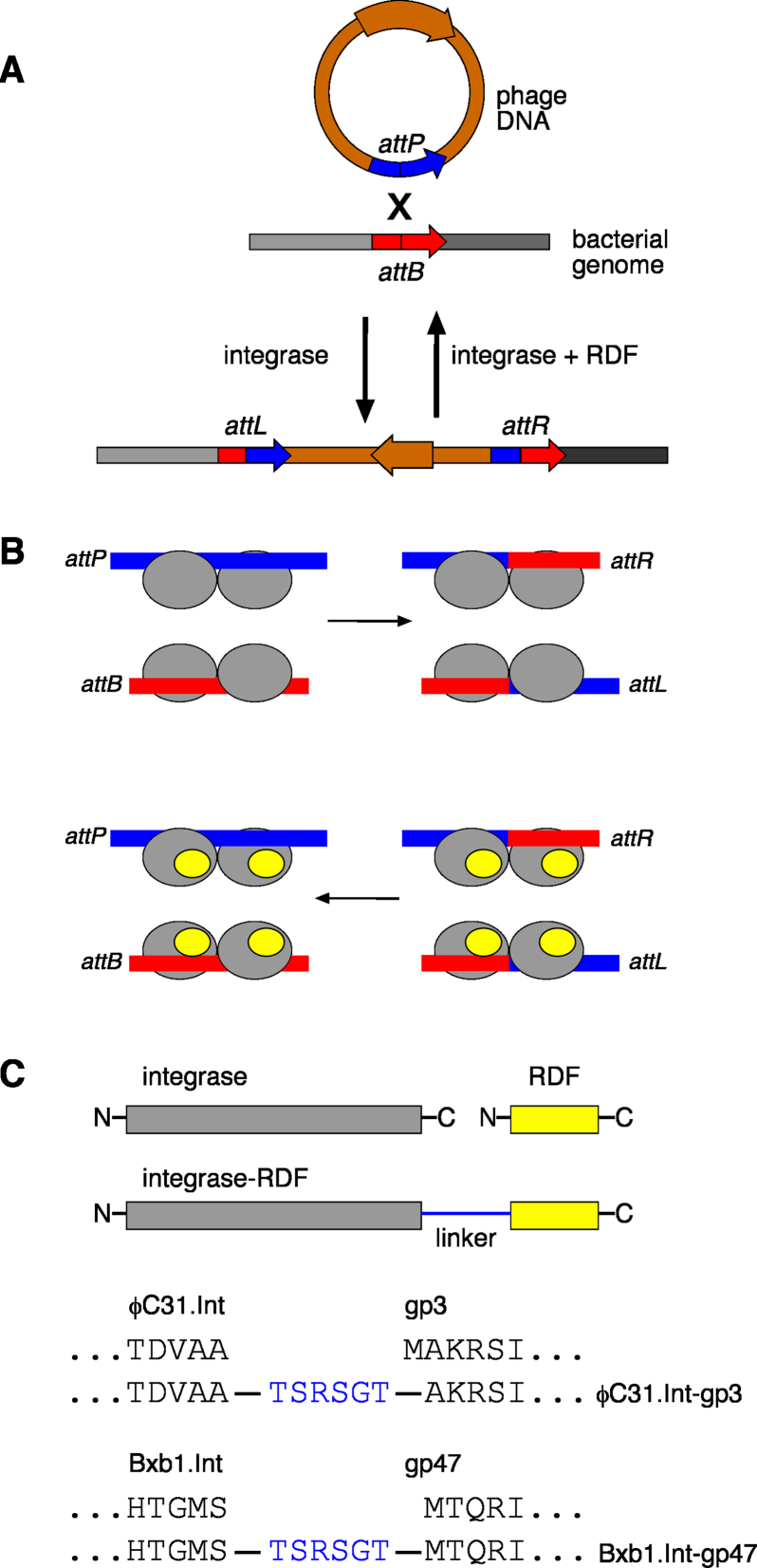
Recombination by serine integrases, and design of integrase–RDF fusion recombinases. (**A**) The biological function of bacteriophage serine integrases. Recombination between a site in the bacteriophage DNA (*attP*) and a site in the bacterial genome (*attB*) results in integration of the phage DNA into the bacterial genome. The integrated phage DNA is flanked by two new recombination sites (*attL* and *attR*). Due to asymmetry within the *att* site sequences, recombination occurs such that the phage DNA is integrated in a specific orientation (indicated by the brown arrow). The ‘reverse’ process, excision of the integrated prophage DNA, is also catalysed by the integrase, but requires a second phage-encoded protein, the recombination directionality factor (RDF). (**B**) Mechanism of integrase-mediated recombination. The *attP* and *attB* sites are each bound by an integrase dimer (grey ovals), and the two dimers then interact to form a synaptic tetramer (not shown). The DNA strands are then broken and rejoined at the centres of the sites to form *attL* and *attR* recombinants. The reverse reaction occurs only in the presence of the RDF (smaller yellow ovals) which binds to integrase and modifies its properties. (**C**) Design of integrase–RDF fusion proteins; linker sequences between the C-terminus of the integrase and the N-terminus of the RDF are as shown (in blue).

Bacteriophage integrases catalyse recombination between an *attP* site in the circularized phage DNA and an *attB* site in the chromosome of the bacterial host cell, leading to an integrated prophage flanked by recombinant *attL* and *attR* sites (2) (Figure 1A). The prophage persists in this integrated state until lysogenic induction, when the integrase promotes recombination between the *attL* and *attR* sites, excising the phage genome as a DNA circle. The phage then re-enters a lytic developmental cycle. Because integrases promote recombination between two sites with different sequences (unlike many other SSRs), the recombinant sites are different from the non-recombinant sites and also each other. These differences can be exploited by the integrase so as to favour integration at lysogeny and excision at lysogenic induction, a property called directionality. Bacteriophage integrases fall into two families according to their protein structures and mechanisms, called tyrosine integrases and serine integrases (3). The two families are mechanistically and evolutionarily unrelated (2,4).

In contrast to tyrosine integrase systems which typically have long *attP* sites and complex accessory protein requirements, the *att* sites for serine integrases are relatively short (∼40–50 bp), and each *att* site binds a dimer of integrase (2) (Figure 1B). Recombination takes place in a synaptic complex where two *att* sites are held together by an integrase tetramer, formed by dimer-dimer interactions. In the absence of any other proteins, a serine integrase catalyses *attP* × *attB* recombination but not the ‘reverse’ *attL* × *attR* recombination. However, another phage-encoded protein called the recombination directionality factor (RDF) makes protein-protein interactions with integrase that transform its properties so that it selectively catalyses *attL* × *attR* recombination (Figure 1B). Directionality is thus switched simply by the presence or absence of RDF. It is hypothesized that binding of RDF to integrase induces protein conformational changes that favour productive synapsis of *attL* and *attR* sites, and disfavour productive *attP* × *attB* synapsis (2). Current experimental evidence suggests that optimal stimulation of *attL* × *attR* recombination by RDF requires interaction of an RDF subunit with each subunit of the tetramer (Figure 1B) (5–7).

Serine integrases have been widely adopted for applications in molecular genetics, biotechnology and synthetic biology because of their directionality, recombination efficiency, and simple DNA sequence requirements (3). Published applications include assembly of long DNA modules (for example, to create arrays of genes encoding metabolic pathway enzymes) (8–10), integration of exogenous DNA modules into cellular genomic DNA and subsequent manipulation of the integrated DNA (1,3,11–13), and construction of genetic logic and memory systems (3,14–20). However, most of these applications have involved just *attP* × *attB* recombination; the additional functionality of *attL* × *attR* recombination has been much less exploited. There are several reasons for this. First, relatively few RDFs have been characterized compared to the large number of known serine integrases, as they are very diverse proteins that are much more difficult to identify than integrases (2,21). Second, *attL* × *attR* recombination requires the presence of integrase, RDF and substrate in appropriate stoichiometric ratio, complicating its use *in vivo* or *in vitro*. Third, *attL* × *attR* recombination efficiency (conversion of substrate to products) is usually lower than for *attP* × *attB* recombination. When no RDF is present, a typical integrase recombines *attP* and *attB* sites efficiently and has no detectable reverse activity on the *attL, attR* products. The presence of RDF switches integrase to favour *attL* × *attR* recombination but does not completely block *attP* × *attB* recombination, either because of the intrinsic properties of the system or because some integrase molecules are not bound by RDF (5). Conversion of *attL* × *attR* substrates may therefore be incomplete.

Here, we aimed to optimize integrase–RDF interaction and stoichiometry by linking the two proteins in a single polypeptide. We hypothesized that such fusion proteins would promote high-efficiency, highly directional *attL* × *attR* recombination, and would thus expand the utility of serine integrase-based systems.

## MATERIALS AND METHODS

### Plasmids and DNA

Plasmids for *in vitro* analysis of integrase activity, containing two *att* sites, were constructed by inserting synthetic double-stranded *att* site oligonucleotides into pFM122, using procedures as described (22,23) (Supplementary Figure S1, Tables S1 and S2). The plasmids are named here according to the type of their *att* sites; for example, pϕC31LRX has ϕC31 *attL* and *attR* sites (the ‘X’ is to distinguish these *in vitro* substrates from the *in vivo* substrates described below). In all the *in vitro* substrates used here, the *att* sites are oriented ‘head to tail’, such that recombination results in resolution of the substrate plasmid into two smaller circles (see Figure 3B). Supercoiled plasmid DNA was purified from transformed *Escherichia coli* DH5 cells, using a Qiagen HiSpeed Midi kit according to the manufacturer's instructions. DNA concentrations were estimated by measuring absorbance at 260 nm.

**Figure 2.**
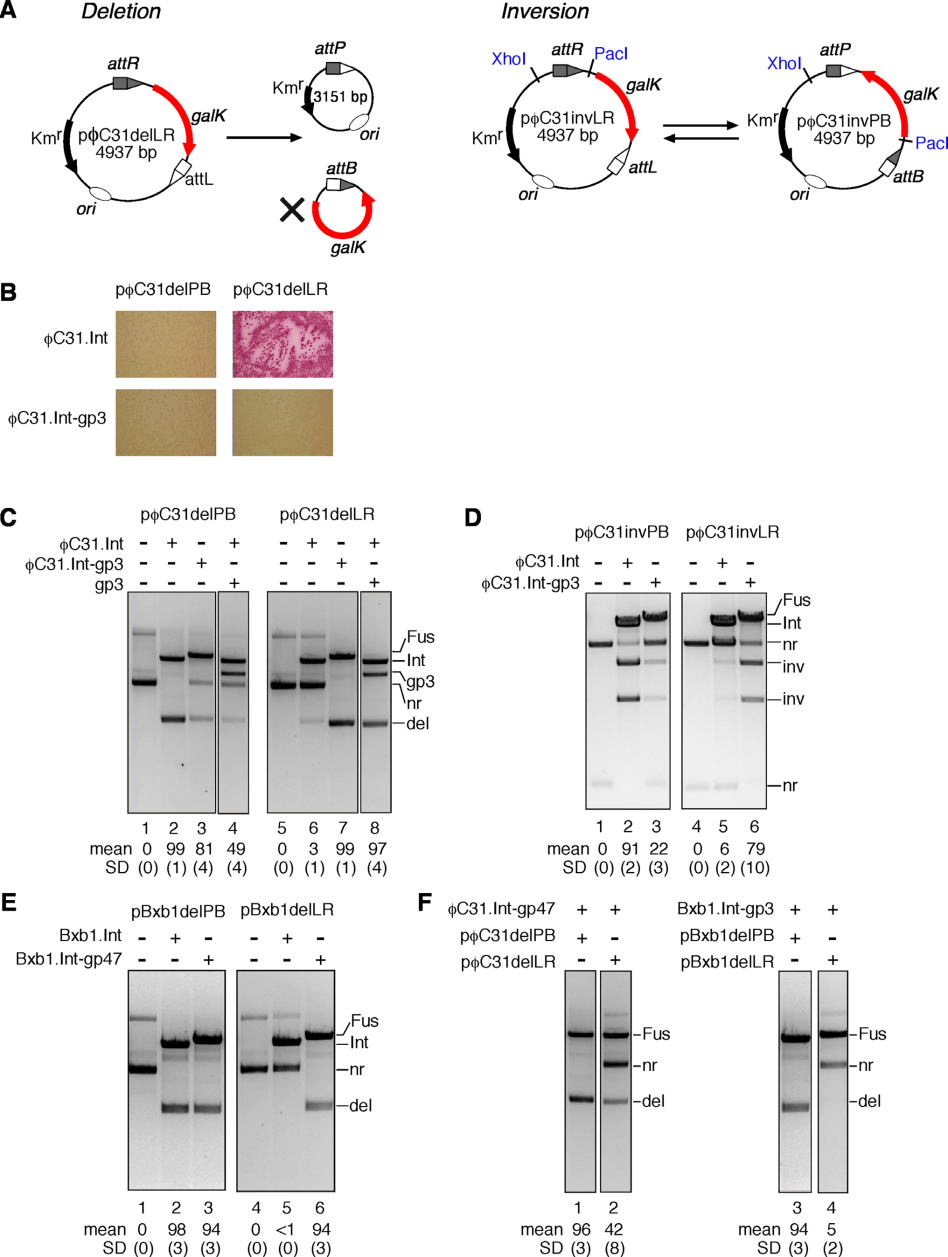
Recombination by integrase–RDF fusion proteins *in vivo*. (**A**) The *in vivo* assay. Left panel: recombination of a deletion substrate. The product *galK*-containing DNA circle (marked with an X) has no origin of replication, so is lost as the cells multiply; this results in a colony color change on MacConkey galactose medium, which can be assessed by visual inspection (24). Right panel: recombination of an inversion substrate flips the orientation of the *galK* gene, which does not alter the size of the plasmid (or *galK* phenotype) but changes the sizes of the restriction digest products. (**B**) Recombination of *attP* × *attB* and *attL* × *attR* deletion substrates (pϕC31delPB and pϕC31delLR respectively) by ϕC31 integrase and the integrase–RDF fusion protein ϕC31.Int-gp3 *in vivo*; colony color assay. (**C**–**F**) Analysis of *in vivo* recombination product DNA. Cells contained the plasmids indicated above the gel images. Miniprep DNA recovered from cells was treated with ScaI, linearizing the recombinase expression plasmid(s) but not the substrate or product plasmids, prior to 1.2% agarose gel electrophoresis. Samples in D were also treated with XhoI and PacI, to reveal inversion products. Expression plasmid linear bands are labelled Int (integrase), Fus (fusion protein), gp3 (RDF). Other bands are labeled nr (non-recombinant, i.e. substrate), inv (inversion product), del (deletion product). Mean extent of recombination and standard deviation (%) from quantitation of triplicate experiments are given below each lane.

*In vivo* test substrates for detecting ϕC31 integrase or integrase–RDF fusion protein activity were made from pFM141 (derived from pDB35; (24)), which contains a pSC101 origin of replication, a kanamycin resistance gene, and a *galK* gene flanked by unique restriction enzyme sites allowing for insertion of the *att* recombination sites as double-stranded synthetic oligonucleotides (Supplementary Figure S1, Table S1). These plasmids are named as described above, with ‘del’ and ‘inv’ indicating predicted outcome of recombination, which depends on the relative orientation of the two *att* sites (see Figures 1A and 2A). For example, pϕC31delLR has ϕC31 *attL* and *attR* sites flanking *galK*, oriented ‘head-to-tail’ such that recombination results in deletion of the *galK* segment, whereas the *att* sites in pϕC31invLR are ‘head-to-head’, so recombination results in inversion of the orientation of the *galK* segment. Similar test substrates for Bxb1 integrase were made by inserting *att* site oligonucleotides into pMS183Δ, a variant of pFM141 with a different set of cloning sites.

Plasmids for constitutive expression of integrases and integrase–RDF fusion proteins in *E. coli* were constructed by inserting protein-coding DNA fragments between unique NdeI and Asp718 sites in pMS140, a low-level expression vector with a pMB1 origin of replication (25). Plasmids for constitutive expression of the RDFs (gp3 for ϕC31 integrase, and gp47 for Bxb1 integrase) were made similarly by inserting appropriate coding sequences into pEK76, which has a p15a (pACYC184) origin (25). The ϕC31 integrase sequence (including a C-terminal His-tag) was derived from pARM010 (23,26) and the gp3 sequence was cloned from pEY301 (6). Codon-optimized sequences of Bxb1 integrase and its RDF gp47 were from GeneArt (Invitrogen).

A DNA sequence encoding the ϕC31 integrase–RDF fusion protein (ϕC31.Int-gp3) was made by joining the coding sequences for ϕC31 integrase and gp3 via an 18 bp linker sequence (ACTAGTAGATCTGGTACC) which contains recognition sequences for the restriction endonucleases SpeI, BglII and Acc65I (in that order), and is translated to a 6-amino acid peptide (TSRSGT) between the C-terminal residue of ϕC31 integrase and the second amino acid residue in gp3 (Figure 1C). The same approach was used to construct coding sequences for the Bxb1 integrase–RDF fusion protein (Bxb1.Int-gp47) and ‘hybrid’ fusion proteins ϕC31.Int-gp47 and Bxb1.Int-gp3. Full sequences of representative plasmids are in Supplementary Table S2.

### Expression and purification of integrases, RDFs and integrase–RDF fusions

Overexpression plasmids for ϕC31 integrase, integrase–RDF fusion proteins, and gp47 were made by cloning coding sequences as described above into pET-28a(+) (Novagen), between the NdeI and XhoI sites. The proteins expressed from these plasmids have an N-terminal hexahistidine tag (MGSSHHHHHHSSGLVPRGSHM followed by integrase amino acid 2), to allow purification via nickel affinity chromatography. For Bxb1 integrase overexpression, the coding sequence with a C-terminal hexahistidine tag (TSHHHHHH) was cloned into pSA1101 (24) between the NdeI and Acc65I sites. pEY301 (6) was used for the overexpression of gp3. All these plasmids confer kanamycin resistance.

All the proteins used in this work were purified by essentially the same protocol. The strain BL21(DE3)pLysS was transformed with the relevant overexpression plasmid. Starter cultures (2 × YT-broth (10 ml) containing kanamycin (50 μg/ml) and chloramphenicol (25 μg/ml)) were inoculated from single transformant colonies and grown overnight at 37°C. An aliquot of starter culture (4 ml) was used to inoculate pre-warmed 2 × YT-broth containing kanamycin and chloramphenicol (400 ml, in 2-l conical flasks). The cultures were incubated at 37°C with shaking (250 rpm) until an optical density of 0.5–0.6 at 600 nm was reached. The cultures were then cooled down rapidly to 20°C, and integrase expression was induced by adding isopropyl-β-d-thiogalactopyranoside (IPTG) (final concentration 0.5 mM). The cultures were then grown for 16 h at 20°C, shaking at 200 rpm. Cells were harvested by centrifugation at 4°C. The resulting cell pellet (∼2 g) was washed in 100 ml 20 mM Tris–HCl (pH 7.5), 10 mM MgCl_2_, and the pellet was collected by centrifugation. The washed pellet was resuspended in 25 ml of a buffer containing 20 mM sodium phosphate (pH 7.4), 1 M NaCl, 1 mM dithiothreitol (DTT), 50 mM imidazole, 1.2 mM phenylmethylsulfonyl fluoride (PMSF) and 1% (v/v) ethanol. The suspension was cooled in ice, and the cells were broken by sonication (Vibra-Cell instrument, Sonics and Materials Inc.); 40% amplitude; 3 × 20-s bursts). The suspension was centrifuged for 30 min at 4°C, 48 000 g. The supernatant was collected and passed through a 0.22 μm filter. The solution containing the soluble protein was then purified by metal affinity chromatography using a 1 ml HisTrap FF pre-packed column (GE Healthcare). The column was equilibrated with 10 column volumes of Buffer A (20 mM sodium phosphate (pH 7.4), 1 M NaCl, 1 mM dithiothreitol (DTT) and 50 mM imidazole), at a constant flow rate of 1 ml/min, prior to loading the protein solution, also in Buffer A. Buffer A (25–30 ml) was then passed through the column to wash off unbound proteins and other cellular components. Following establishment of a steady baseline (absorbance at 260 and 280 nm), bound protein was eluted with Buffer B (Buffer A, but with 500 mM imidazole), increasing in a 0–100% linear gradient over 25 min. Purity of fractions was assessed by SDS-polyacrylamide gel electrophoresis. Selected fractions were dialysed against 1 l of Buffer C (25 mM Tris–HCl (pH 7.5), 1 mM DTT, 1 M NaCl and 50% glycerol), for at least 6 h, then in a further 1 l of fresh Buffer C for a further 6 h, and stored at –20°C. Protein concentrations were estimated by measurement of absorbance at 280 nm and/or comparison with standards on discontinuous SDS-polyacrylamide gels stained with Coomassie Brilliant Blue G-250. The purified proteins were used without removing the His tags.

### *In vivo* recombination reactions

Assays for integrase-mediated recombination in *E. coli* were modified from the method described (for zinc-finger recombinases) in Akopian *et al.* (27). Briefly, the *galK* mutant *E. coli* strain DS941 was first transformed with an appropriate test substrate plasmid. Competent cells were prepared from the substrate plasmid-containing strain, and these were transformed with an integrase-expressing plasmid, or co-transformed with integrase- and RDF-expressing plasmids. Transformants were selected on MacConkey-galactose indicator plates (MacConkey agar base (Difco) supplemented with 1% galactose, kanamycin (50 μg/ml; to select for the substrate plasmid and its recombination product), ampicillin (50 μg/ml; to select for the integrase expression plasmid), and chloramphenicol (25 μg/ml, to select for the RDF expression plasmid when necessary). When the substrate plasmid has *att* recombination sites arranged such that recombination causes deletion of the *galK* gene, pale-coloured (*galK^−^*) colonies on the indicator plates indicate recombination proficiency, whereas red (*galK^+^*) colonies indicate lack of recombination; see Figure 2A and B.

To determine the extent of *in vivo* recombination more accurately, plasmid DNA was isolated. The cells on the MacConkey-galactose agar plates were recovered by suspending them in L-broth (1 ml per plate). An aliquot of the suspension (1 μl) was used to inoculate L-broth (10 ml), and this culture was incubated overnight at 37 °C with kanamycin selection. Plasmid DNA was prepared using a Qiagen miniprep kit. Samples of the plasmid DNA were digested with appropriate restriction enzymes, then analysed by 1.2% agarose gel electrophoresis. All samples were treated with ScaI, which linearizes the recombinase expression vectors but not the substrate or recombinant plasmids. Products from cells containing inversion substrates were additionally treated with XhoI and PacI to produce distinct DNA fragments from non-recombinant and recombinant molecules. Quantitation of bands on the gels was as described (5). All experiments were performed in triplicate, starting with three separate transformations of the integrase-expressing plasmid as described above.

### *In vitro* recombination reactions and product analysis

Integrases, integrase–RDF fusion proteins, and gp3 were each diluted at 0°C in a buffer containing 25 mM Tris–HCl (pH 7.5), 1 mM DTT, 1 M NaCl and 50% (v/v) glycerol. Dilutions were stored at –20°C. In a typical recombination reaction, diluted integrase (∼4 μM, 4.5 μl) was added to a solution (40 μl) containing substrate plasmid DNA (20 μg/ml), bovine serum albumin (100 μg/ml), 50 mM Tris–HCl (pH 7.5), 5 mM spermidine, and 0.1 mM EDTA. The final integrase concentration was thus approximately 400 nM. The sample was incubated at 30°C for 60 min. For reactions in the presence of gp3, equal volumes of integrase (∼8 μM) and gp3 (in the same buffer; ∼8 μM) were mixed thoroughly and kept on ice for 15 min, then 4.5 μl of this mixture (containing ∼4 μM of each protein) was added to the reactions. Similar protocols were followed in reactions involving mixtures of integrase and integrase–RDF fusion proteins; see text for details. Reactions were terminated by heating at 80°C for 10 min. For analysis by restriction enzyme digestion, aliquots of the reaction mixture (30 μl) were mixed with B103 buffer (90 mM Tris–HCl pH 7.5, 20 mM MgCl_2_; 28 μl) prior to addition of NruI (New England Biolabs; 2 μl, 20 units). The samples were then incubated at 37°C for 2 h. Loading buffer (25 mM Tris–HCl (pH 8.2), 20% (w/v) Ficoll, 0.5% sodium dodecyl sulfate, 5 mg/ml protease K and 0.25 mg/ml bromophenol blue; 7.5 μl) was added to each sample, and then the products were separated by agarose gel electrophoresis and visualized as described (28,29). Digital images of ethidium-stained gels were recorded using a BioRad GelDoc apparatus, and are shown in reverse contrast.

### *In vitro* gene editing and product analysis

The methods for the cassette exchange experiments were as described (8) (see also Figure 6). Recombination between a Cm^R^*/ccdB* fragment with *attR* sites at each end (4 nM) and *attL* sites in p(BEIZY) (12 nM) was promoted *in vitro* by a mixture of ϕC31 integrase (200 nM) and gp3 (200 or 400 nM), or by ϕC31.Int-gp3 fusion protein (200 nM). Reactions (conditions as described in the previous section) were for 1, 2 and 18 h. The reaction products were used to transform *E. coli* DB3.1 cells by electroporation; transformants were selected with ampicillin and chloramphenicol on L-agar plates. The numbers of colonies recovered were counted and used as an assessment of the efficiency of the integrase-catalysed *in vitro* reactions. The colonies from each treatment were then pooled, miniprep DNA was prepared, DNA samples were digested with PstI, and the digested DNA was analysed by 1.2% agarose gel electrophoresis to reveal the proportion of plasmids with correct cassette replacement.

## RESULTS

### Design of integrase–RDF fusion proteins, and activity *in vivo*

We chose to focus our studies on two well-characterized serine integrases for which RDFs have been identified; ϕC31 integrase (605 amino acids) from a *Streptomyces* phage, and Bxb1 integrase (500 amino acids) from a *Mycobacterium* phage. RDFs are known to interact directly with their cognate integrase protein (2,6,30,31), but the interacting surfaces of the proteins remain unidentified. Current hypotheses based on mutational analysis and crystallographic structures of the C-terminal part of a *Listeria innocua* phage (LI) integrase bound to *att* site DNA (32–34) propose that the interactions involve (or are close to) a coiled-coil domain of the protein. We noted that the extreme C-terminus of the LI integrase lies quite near the coiled-coil domain in the solved structures, so an RDF moiety attached here via a short flexible peptide linker might be able to interact with the coiled-coil domain of its own fusion protein molecule. However, the LI integrase is the only one whose structure in complex with DNA has been solved, and there are as yet no structures of the integrase–RDF interaction for any system. It was therefore not possible to model integrase–RDF interactions of our chosen systems in detail, so we decided to adopt a simple strategy, attaching the RDF to the C-terminus of integrase via a flexible linker peptide.

**Figure 3.**
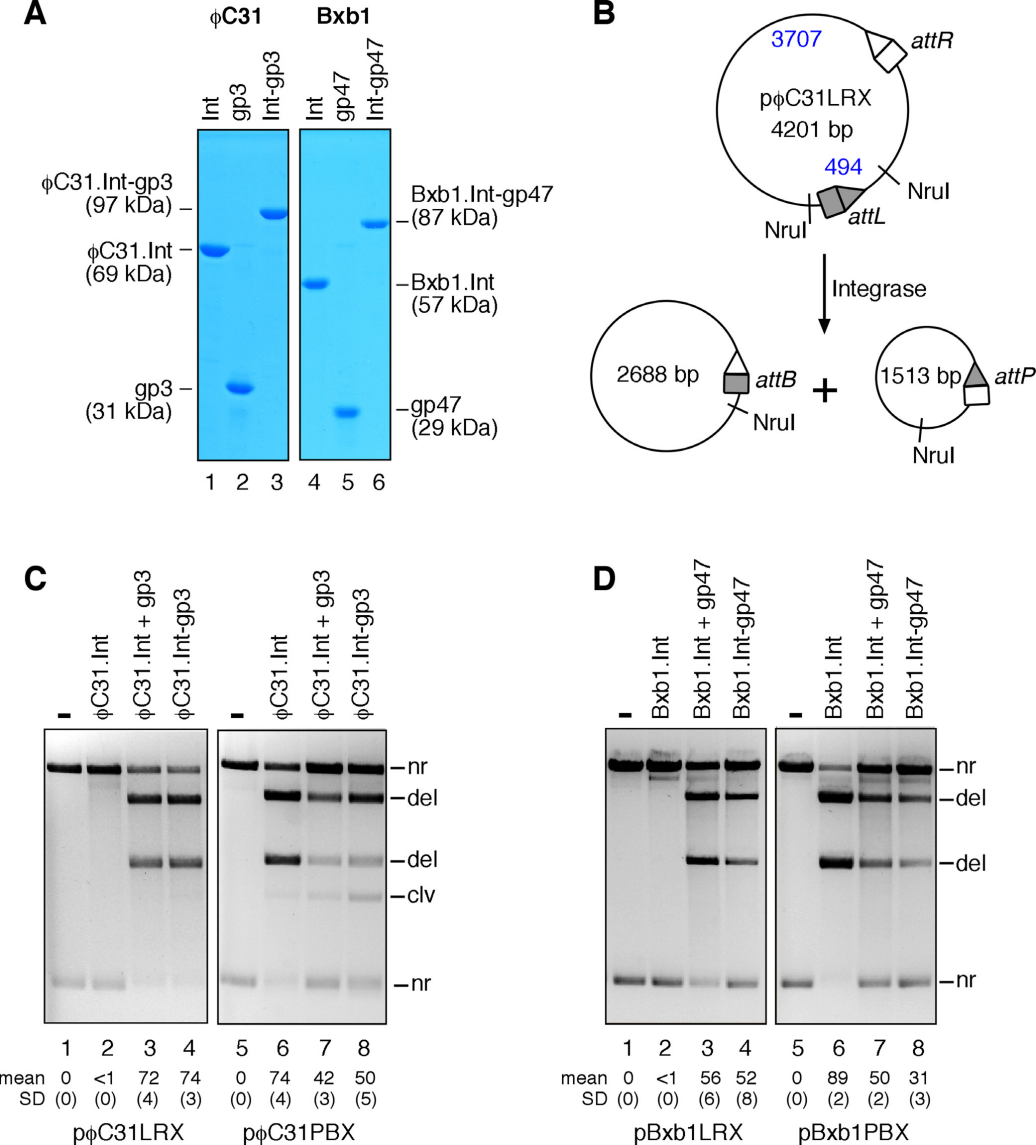
Recombination by integrase–RDF fusion proteins *in vitro*. (**A**) SDS-PAGE analysis of purified ϕC31 and Bxb1 integrases, RDFs, and fusion recombinases. Lane 1, ϕC31.Int; lane 2, gp3; lane 3, ϕC31.Int-gp3; lane 4, Bxb1.Int; lane 5, gp47; lane 6, Bxb1.Int-gp47. (**B**) Scheme illustrating the *in vitro* recombination assay (substrate plasmid pϕC31LRX shown; other substrates are similar). Reaction products were digested with NruI prior to 1.2% agarose gel electrophoresis. (**C**) Recombination of ϕC31 *attL* × *attR* and *attP* × *attB* plasmid substrates by ϕC31 integrase with or without gp3, or by the fusion protein ϕC31.Int-gp3, as indicated above the lanes. The band labelled clv derives from double-strand cleavage of some substrate by integrase at the upper (unshaded) site as in Figure 2B. For the pϕC31PBX reactions (where this band is most obvious; lanes 7 and 8), this site is *attP*. (**D**) Recombination of Bxb1 *attL* × *attR* and *attP* × *attB* plasmid substrates by Bxb1 integrase with or without gp47, or by the fusion protein Bxb1.Int-gp47. In parts C and D, the final integrase, fusion protein, and RDF concentrations were all 400 nM. The mean extent of recombination and standard deviation (%) from quantitation of triplicate experiments are given below each lane.

The 244-amino acid ϕC31 RDF gp3 (6) was attached to the C-terminus of ϕC31 integrase via a 6-amino acid flexible linker, making the 854-amino acid fusion protein ϕC31.Int-gp3 (Figure 1C). The activity of ϕC31.Int-gp3 was tested using a standard assay in which *E. coli* cells are first transformed with a test plasmid containing two recombination sites, and then a plasmid expressing the recombinase (24,35). Recombination of the test plasmid results in the deletion of a *galK* marker gene (Figure 2A, left panel) and a consequent change of colony colour on indicator plates. The substrate plasmid pϕC31delLR, containing ϕC31 *attL* and *attR* sites, was not recombined by ϕC31 integrase (red colonies), as expected in the absence of the RDF gp3 (Figure 2B), whereas it was recombined efficiently by the fusion protein ϕC31.Int-gp3 (pale-coloured colonies). A similar plasmid with *attP* and *attB* sites flanking *galK* (pϕC31delPB) was recombined both by ϕC31 integrase and ϕC31.Int-gp3. Plasmid DNA from the colonies on the plates was then analysed by agarose gel electrophoresis (Figure 2C). In accord with the observed colony colours, the *attL* × *attR* test substrate pϕC31delLR was observed to be mostly recombined in cells expressing ϕC31.Int-gp3 (Figure 2C, lane 7), whereas it was mostly unrecombined in cells expressing ϕC31 integrase (Figure 2C, panel 6). The low but reproducibly observable level of recombination of this substrate by ϕC31 integrase is curious, because *in vitro*, ϕC31 integrase does not promote *attL* × *attR* recombination at all; for example, see Figure 3. We suspect that an unidentified endogenous *E. coli* protein can substitute weakly for gp3 to permit *attL* × *attR* recombination. As expected, the *attP* × *attB* substrate pϕC31delPB was fully recombined by unmodified ϕC31 integrase (Figure 2C, lane 2), but recombination in cells expressing the integrase–RDF fusion protein ϕC31.Int-gp3 (Figure 2C, lane 3), or integrase and gp3 separately (Figure 2C, lane 4) was incomplete, showing that the presence of the gp3 moiety of the fusion protein or gp3 itself has an inhibitory effect.

We carried out another set of experiments using similar test substrates in which the *att* sites flanking *galK* are in inverted repeat (head-to-head) rather than direct repeat. Recombination of these substrates does not delete the *galK* segment, but instead inverts its orientation relative to the remainder of the plasmid. The recombinant plasmid can potentially recombine again, restoring the original ‘non-recombinant’ DNA configuration (Figure 2A, right panel). Restriction enzyme digestion followed by gel electrophoresis of plasmid DNA isolated from *E. coli* cells reveals the relative amounts of non-recombinant and recombinant test plasmids. The *attL* × *attR* substrate pϕC31invLR was recombined efficiently (79%) in cells expressing the fusion protein ϕC31.Int-gp3 (Figure 2D, lane 6), but this plasmid was poorly recombined (6%) by ϕC31 integrase itself (Figure 2D, lane 5). In contrast, the *attP* × *attB* plasmid pϕC31invPB was recombined efficiently (91%) by ϕC31 integrase but less efficiently (22%) by ϕC31.Int-gp3 (Figure 2D, panels 1–3). The gp3 moiety of the fusion protein thus stimulates *attL* × *attR* recombination but inhibits *attP* × *attB* recombination, so that at equilibrium most of the inversion substrate is in the configuration with *attP* and *attB* sites, regardless of whether the starting plasmid had *attP* and *attB*, or *attL* and *attR* sites.

To test the general applicability of the integrase–RDF fusion strategy, we used analogous methods to construct a fusion protein of Bxb1 integrase and its RDF gp47 (Bxb1.Int-gp47; Figure 1C). The Bxb1 and ϕC31 RDFs gp47 and gp3 are unrelated proteins. Like its ϕC31 counterpart, Bxb1.Int-gp47 promoted recombination of both *attL* × *attR* and *attP* × *attB* substrates *in vivo*, whereas Bxb1 integrase itself promoted recombination of the *attP* × *attB* substrate only (Figure 2E). Inhibition of *attP* × *attB* recombination by the gp47 moiety of the Bxb1.Int-gp47 fusion protein was not observed in this *in vivo* experiment.

### Specific effect of the RDF moiety on fusion protein activity

It was possible that the altered recombination properties of the fusion proteins were caused by some non-specific effect of the integrase C-terminal extension, rather than by specific integrase–RDF interactions. To test this idea, we made ‘RDF-swapped’ versions of both ϕC31 and Bxb1 integrases; that is, we linked gp47 to ϕC31 integrase (ϕC31.Int-gp47), and gp3 to Bxb1 integrase (Bxb1.Int-gp3). Each of these hybrid proteins fully recombined the *attP* × *attB* substrate with sites recognized by its integrase moiety, but had lower (ϕC31.Int-gp47) or zero (Bxb1.Int-gp3) activity on the corresponding *attL* × *attR* substrate (Figure 2F). We conclude that full stimulation of the integrase to perform efficient *attL* × *attR* recombination requires C-terminal fusion to its own RDF.

### *In vitro* recombination activity of integrase–RDF fusion proteins

Experiments with integrase–RDF fusion proteins *in vivo* might give misleading results if the RDF is present in the cells as a free protein (due to proteolytic cleavage of the fusion protein, or initiation of translation within the fusion protein reading frame). To eliminate such possibilities, we analysed the activities of the fusion proteins in an *in vitro* assay, where recombination of a plasmid substrate containing a pair of *att* sites can be detected by restriction enzyme digestion and gel electrophoresis of the products. SDS-PAGE analysis of purified ϕC31.Int-gp3 showed a single polypeptide of the predicted molecular weight (97 kDa; Figure 3A). This fusion protein promoted efficient (74%) recombination of the *attL* × *attR* substrate pϕC31LRX (Figure 3C, lane 4). The extent of recombination was as high as when a mixture of ϕC31 integrase and gp3 was used (Figure 3C, lane 3). Unmodified ϕC31 integrase (in the absence of gp3) did not promote any recombination of this substrate (Figure 3C, lane 2). In contrast, ϕC31 integrase promoted efficient (74%) recombination of the *attP* × *attB* substrate pϕC31PBX, but recombination by ϕC31.Int-gp3 was less efficient (50%); again, the extent of the reaction with the ϕC31.Int-gp3 fusion protein was similar to that observed with integrase plus gp3 (Figure 3C, lanes 6–8). These results confirm that the ϕC31.Int-gp3 fusion protein does indeed promote *attL* × *attR* recombination as a single polypeptide, and that it has reduced *attP* × *attB* activity.

We also expressed and purified the Bxb1.Int-gp47 fusion protein (Figure 3A). Like its ϕC31 equivalent, Bxb1.Int-gp47 promoted *attL* × *attR* recombination *in vitro*, in contrast to Bxb1 integrase itself which was inactive on this substrate (Figure 3D; compare lanes 4 and 2). Similarly, recombination of an *attP* × *attB* substrate by the fusion protein was reduced relative to unmodified Bxb1 integrase (Figure 3D; compare lanes 8 and 6).

### The integrase–RDF regulatory interaction is intramolecular

**Figure 4.**
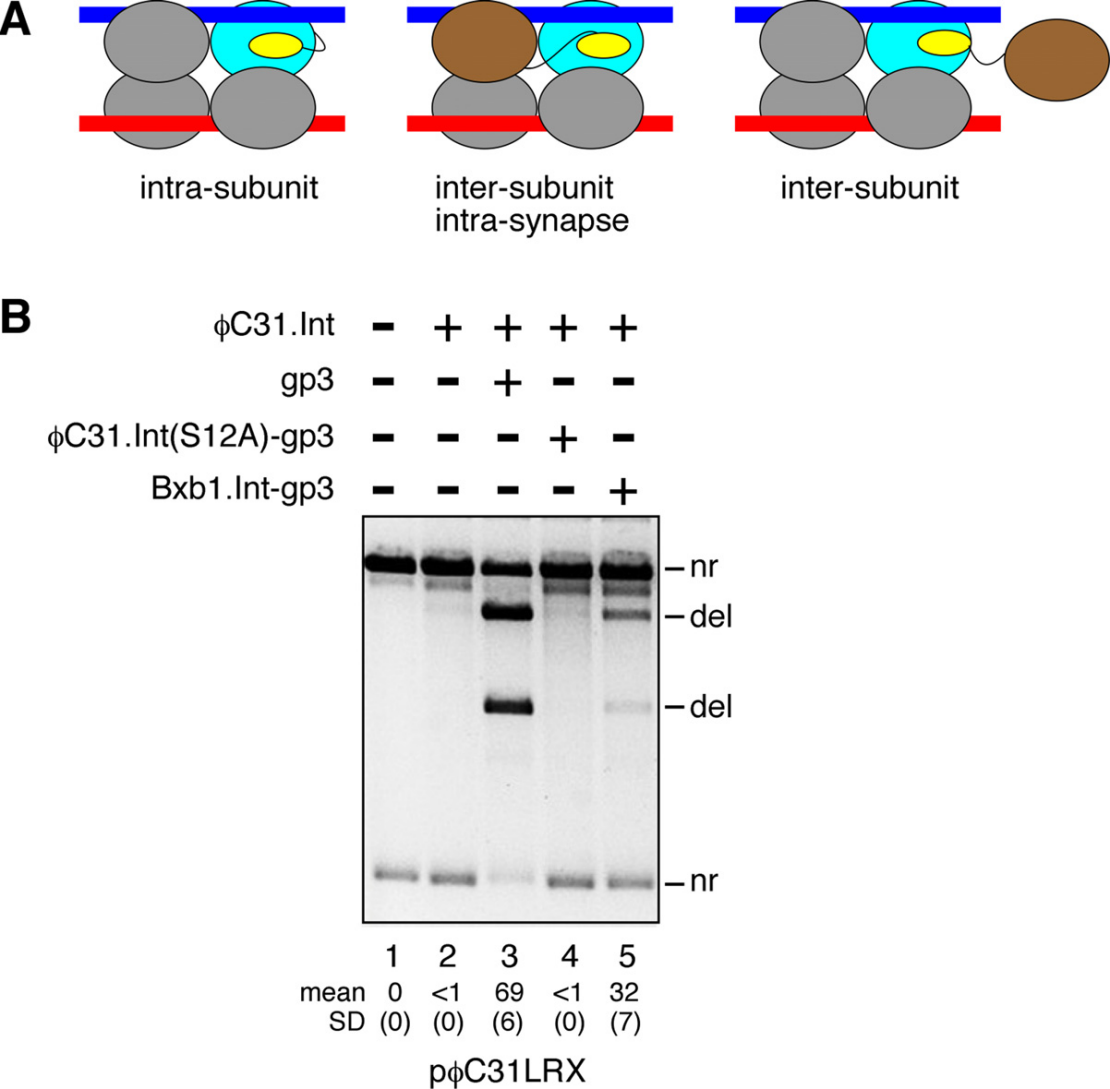
Stimulation of integrase activity by RDF is intramolecular. (**A**) Hypothetical intra-subunit or inter-subunit activating interactions of the RDF and integrase moieties of an integrase–RDF fusion protein. The diagrams represent the intermediate synaptic complex formed when two integrase-bound *att* sites interact. Only one RDF moiety is shown in each diagram, for clarity. The stimulated integrase subunit is in blue, and the subunit donating the RDF moiety (where different) is in brown. (**B**) ϕC31 integrase (400 nM) was added to pϕC31LRX substrate and the mixture was incubated at room temperature for 5 min to allow binding and full occupation of the sites; then gp3 (lane 3), ϕC31.Int(S12A)-gp3 (lane 4), or Bxb1.Int-gp3 (lane 5) was added. The mixture was then incubated for 1 h at 30°C, and the products were detected as in Figure 3. Mean extent of recombination and standard deviation (%) from quantitation of triplicate experiments are given below relevant lanes.

Our working hypothesis was that the RDF moiety of the fusion protein stimulates *attL* × *attR* recombination by binding to its attached integrase moiety (Figure 4A, left panel). However, it was also possible that activation involves interaction of the RDF moiety of one fusion protein subunit with the integrase moiety of another subunit. Figure 4A illustrates two such inter-subunit scenarios; an RDF-donor subunit could be part of the synaptic complex (the intermediate leading to strand exchange) (Figure 4A, centre panel), or it could be an ‘extra’ subunit which is not part of the synaptic complex (Figure 4A, right panel). We performed an experiment *in vitro* to test for the latter scenario. The fusion protein ϕC31.Int(S12A)-gp3 has a mutation of the catalytic serine residue S12 to alanine and is thus inactive (26), but it could donate its RDF (gp3) moiety to stimulate *attL* × *attR* recombination by unmodified integrase. Unmodified ϕC31 integrase was added first to *attL* × *attR* substrate plasmid, so that it would fully occupy the *att* sites. When gp3 was then added to the mixture, efficient recombination (69%) was observed (Figure 4B, lane 3), but recombination was only barely detectable (<1%) when ϕC31.Int(S12A)-gp3 was added (Figure 4B, lane 4). The gp3 moiety of the fusion protein is thus unavailable for stimulatory inter-subunit interactions with the pre-bound unmodified integrase.

A simple explanation of this result is that the gp3 moiety of the fusion protein is sequestered by binding tightly to its linked integrase moiety. To test this hypothesis, we used a different fusion protein, Bxb1.Int-gp3, comprising Bxb1 integrase linked to the ϕC31 RDF gp3. This protein (like Bxb1 integrase itself) does not bind to or recombine ϕC31 *att* sites (data not shown). Bxb1 integrase and gp3 are from unrelated bacteriophages, so we did not expect the two parts of this fusion protein to interact strongly. When Bxb1.Int-gp3 was added to pre-assembled complexes of the ϕC31 *attL* × *attR* substrate with ϕC31 integrase, it stimulated recombination (Figure 4B, lane 5). The gp3 moiety of this protein is thus free to make inter-subunit interactions, unlike that of the ‘all-ϕC31’ fusion protein.

In summary, these results show that ‘extra’ subunits (not bound to the *att* sites) are not involved in stimulation of *attL* × *attR* recombination activity by the fusion proteins. The simplest explanation of our observations is that the integrase and RDF parts of each fusion protein subunit interact with each other (as in the left-hand panel of Figure 4A).

### The integrase–RDF linker

**Figure 5.**
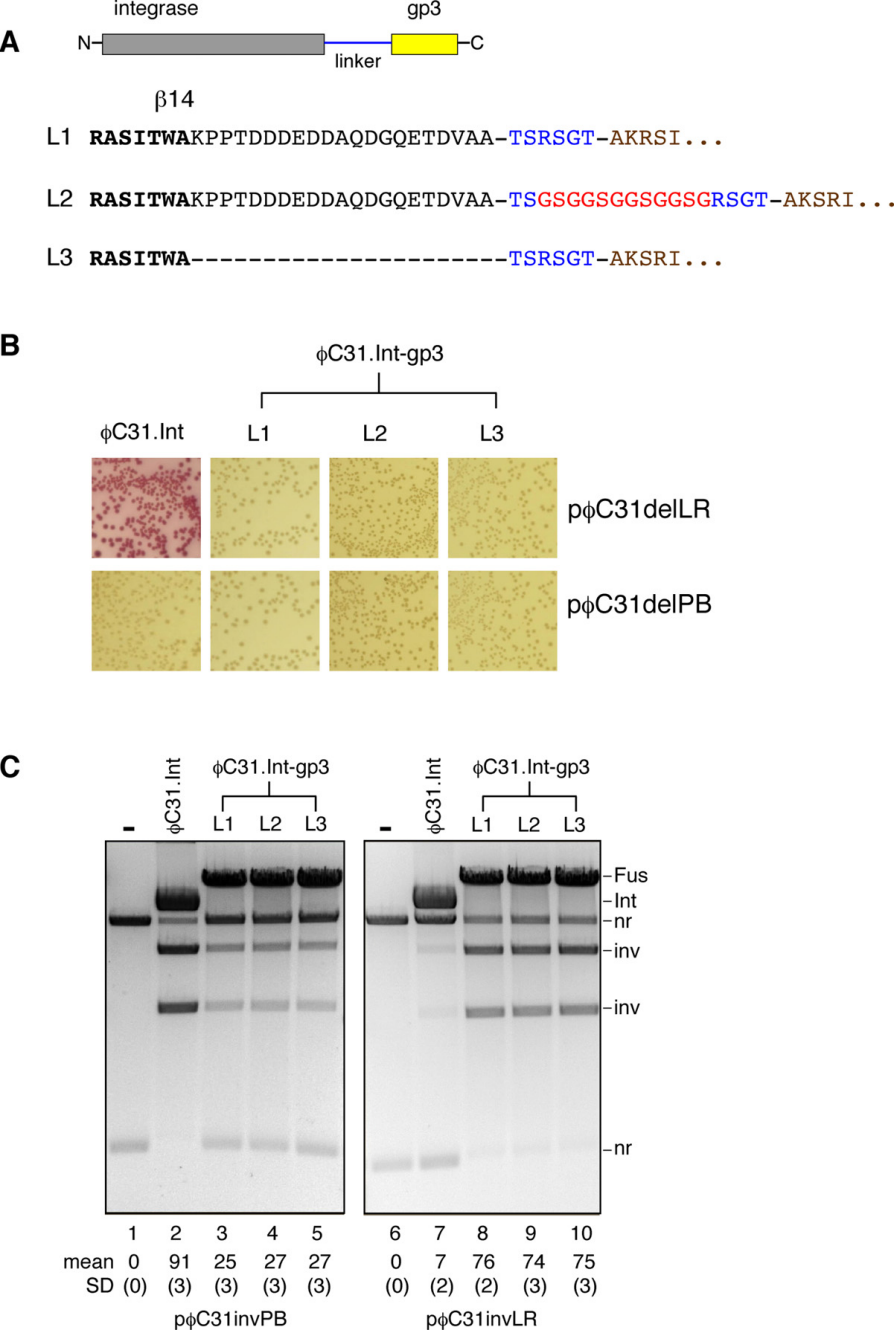
Variation of the integrase–RDF linker. (**A**) Variant linkers between the integrase and gp3 moieties of the fusion recombinase ϕC31.Int-gp3. Linker sequence is in blue (and red showing the flexible (GSG)_4_ sequence); gp3 sequence (starting at gp3 residue 2) is in brown. (**B**) Recombination of a deletion substrate in *E. coli* by the variant-linker fusion proteins. The assay is as shown in Figure 2A and B. (**C**) Products from inversion substrates in *E. coli* were analysed by restriction enzyme digestion and gel electrophoresis, as described in the legend for Figure 2D. Mean extent of recombination and standard deviation (%) from quantitation of triplicate experiments are given below each lane.

The length and nature of the linker peptide connecting the integrase and RDF moieties might be expected to be important factors affecting the properties of the fusion proteins. The linkers used in our first fusions (described above) might have been fortuitously functional, but they might also be sub-optimal. We therefore made and tested fusion proteins with two altered linker designs (Figure 5A; see also Figure 1C). The sequence shown in bold in Figure 5A corresponds to the most C-terminal identified structural element (β-strand 14) of the large serine recombinases (33). In ϕC31 integrase this element is followed by a final 21-amino acid sequence which is non-essential (36) and whose function (if any) is unknown. Our original linker (L1) is a 6-amino acid sequence connecting the C-terminus of ϕC31 integrase to the N-terminus of gp3 (minus its initial methionine residue). Linker L2 includes an additional 12-amino acid (GSG)_4_ sequence, reported to be flexible (28,37,38). The third linker L3 is similar to L1 except that the 21 amino acid residues following β-strand 14 have been deleted.

The activities of the three linker variants of the fusion protein ϕC31.Int-gp3 were compared *in vivo*. There were no apparent differences in activity, as indicated by the colony colour assay (Figure 5B); all three proteins were proficient for recombination on both *attP* × *attB* and *attL* × *attR* substrates. Similarly, examination of DNA isolated from cells harbouring inversion substrates showed no differences in activity; all three proteins recombined most of the *attL* × *attR* substrate, but had lower activity on the *attP* × *attB* substrate (Figure 5C), consistent with our earlier results (Figure 3). We conclude that, within the range we tested, linker length and structure are not critical for fusion integrase recombination activity.

### Gene array editing by integrase–RDF fusion recombinase

Integrase–RDF fusion recombinases might have many uses in serine integrase-based methodologies for DNA manipulation, as single proteins substituting for the two separate proteins currently necessary to promote *attL* × *attR* recombination (see Discussion). Here, as a proof of principle, we show how an ordered array of genes assembled by integrase-mediated *attP* × *attB* recombination can subsequently be edited by fusion recombinase-mediated *attL* × *attR* recombination.

**Figure 6.**
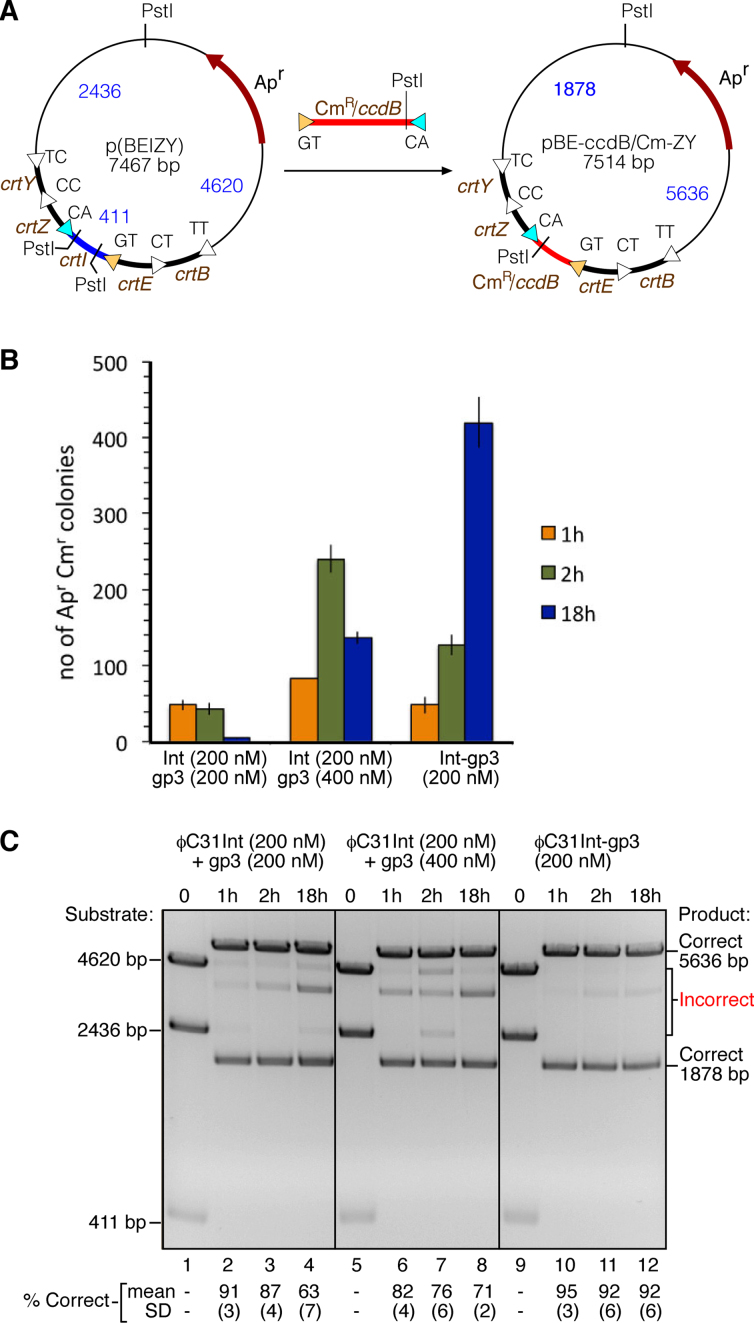
Targeted editing of a gene array by an integrase–RDF fusion recombinase. (**A**) integrase–RDF fusion protein-mediated replacement of the *crtI* gene cassette in plasmid p(BEIZY) with a different cassette (Cm^R^/*ccdB*). p(BEIZY) contains an array of genes *crtB, crtE, crtI, crtZ* and *crtY*, each separated by ϕC31 *attL* sites differing in their central dinucleotide sequence (8). The linear PCR product (red, cartooned above the arrow) contains the Cm^R^*/ccdB* gene cassette (conferring chloramphenicol resistance), flanked by ϕC31 *attR* sites (*attR*-GT and *attR*-CA; yellow and cyan triangles) with central dinucleotides matching the *attL* sites flanking *crtI*. The usefulness of Cm^R^*/ccdB* cassette exchange is described in (8); the properties of the *ccdB* gene are not used in the experiments described here. (**B**) Cassette substitution reactions were carried out *in vitro* with integrase and gp3 or Int-gp3 fusion recombinase, for varying times, as indicated on the figure. Reaction products were used to transform *E. coli* cells, transformants were selected on plates containing ampicillin and chloramphenicol, and colonies were counted. Each bar in the figure represents mean ± standard deviation from three experiments. (**C**) Plasmid DNA was recovered from the pooled *E. coli* colonies (as in B) and digested with PstI. The digest products were separated by agarose gel electrophoresis, and the proportion (%) of correctly assembled plasmids was estimated by quantitation of the bands. Quantitation values and standard deviation from triplicate experiments are given below each lane.

We began with a plasmid (p(BEIZY)) made in previous work (8,9), which contains an array of genes expressing enzymes for a metabolic pathway leading to the carotenoid zeaxanthin. The five carotenoid genes *crtB, crtE, crtI, crtZ* and *crtY* were assembled (in this order) from PCR products using ϕC31 integrase-mediated *attP* × *attB* recombination, so that each gene cassette in p(BEIZY) is flanked by *attL* sites (Figure 6A). The assembly method relied on the specificity of serine integrases for recombination between sites with identical nucleotides in the central 2 bp of the *att* sites; as a result, each *attL* in the array has a different central 2 bp (Figure 6A) (8). In order to replace one of the gene cassettes (*crtI*) with a different sequence, we prepared a PCR product consisting of a gene for chloramphenicol resistance flanked by *attR* sites, with GT and CA central dinucleotides corresponding to the central dinucleotides of the *attL* sites flanking *crtI* in the array. The *crtI* cassette is replaced by the chloramphenicol resistance cassette after two *attL* × *attR* recombination reactions, one between the ‘GT’ sites and the other between the ‘CA’ sites (Figure 6A). A mixture of p(BEIZY) and Cm^R^ PCR product was treated with the ϕC31.Int-gp3 fusion protein, or with integrase plus gp3 (two different concentrations of gp3 were compared). Aliquots of the reactions were stopped after 1, 2 and 18 h, and the products were used to transform *E. coli* cells by a standard protocol (8,9), selecting for ampicillin and chloramphenicol resistance. The numbers of colonies were then counted (Figure 6B). The reactions with the fusion protein and with integrase–gp3 mixtures for 1 or 2 h gave similar transformant colony numbers, but the fusion protein gave more colonies than the mixtures after 18-h reactions, indicating a higher level of cassette exchange. Plasmid DNA was prepared from the pooled colonies from each transformation and analysed by digestion with PstI, to check that the chloramphenicol resistance cassette was correctly inserted into the array (Figure 6C). In all cases most of the plasmid DNA had the expected structure (bands of 5636 and 1878 bp); other fainter bands, indicative of incorrect assembly or post-assembly rearrangements, were evident in all the DNA samples but were notably less abundant in the products of fusion protein-mediated cassette exchange (Figure 6C).

## DISCUSSION

The ϕC31 and Bxb1 integrase–RDF fusion proteins that we constructed promote efficient *attL* × *attR* recombination, in contrast to their parent integrases (which are completely inactive on *attL* × *attR* substrates *in vitro*). The extent of fusion protein-promoted *attL* × *attR* recombination observed under optimal conditions was similar to that of the parent integrase in the presence of a large excess of RDF. In contrast, *attP* × *attB* recombination activity of the fusion proteins was reduced in comparison to the parent integrases. Again, the reduction in activity was similar to that observed when a high concentration of RDF was added to the unmodified integrase. These results, along with our further *in vitro* complementation experiments, imply that the fusion protein acts as a unitary enzyme, with the integrase and RDF parts of each protein subunit binding to each other. The length and sequence of the linker peptide between the two domains of the fusion recombinase might therefore be predicted to be important for optimal interactions, but we did not observe any obvious differences in *attL* × *attR* recombination efficiency for the three linker variants of the ϕC31 integrase-gp3 fusion protein that we tested (Figure 5). We speculate that flexible or disordered regions at the C-terminus of ϕC31 integrase and/or the N-terminus of gp3 contribute to the effective length and flexibility of the inter-domain linker, negating any effects of the relatively small variations that we tested. These results suggest that construction of similar fusion recombinases from other serine integrases will be straightforward as the linker design is not a critical factor.

We successfully created functional fusion recombinases from two different integrases (ϕC31 and Bxb1), and we are confident that the same strategy could be applied to many other serine integrases. It may thus become routine to use two recombinase proteins in serine integrase-based applications, integrase for *attP* × *attB* recombination and integrase–RDF fusion for *attL* × *attR* recombination, rather than using integrase for both but complementing with RDF for the *attL* × *attR* reaction. This dual-recombinase methodology should be advantageous for several reasons. First, comparable expression levels of the two proteins *in vivo* should be easily achievable using identical promoters, which might be switched on or off alternately by a genetic circuit. Second, on expression of the fusion protein, the integrase–RDF stoichiometry is fixed at the predicted optimal value of 1:1 (5), whereas the level of RDF must be adjusted for optimal performance in any experimental system when it is expressed separately (15). Third, RDF-integrase contacts favouring *attL* × *attR* recombination are expected to be maximized by intramolecular linkage of the two proteins. Fourth, fusion of the RDF with the integrase might eliminate problems of RDF toxicity, aggregation or instability in both *in vitro* and *in vivo* applications.

We have shown here how an integrase–RDF fusion recombinase can be used for efficient editing of gene arrays assembled by SIRA methodology (8) (Figure 6). We envisage many similar applications, where the fusion recombinase is used for subsequent manipulations of products of *attP* × *attB* recombination which contain DNA components bounded by *attL* and/or *attR* sites. integrase–RDF fusion recombinases may prove to be especially useful for genetic switches, circuits, logic and memory systems (14–20). For example, an invertible DNA segment in *PB* orientation (that is, bounded by *attP* and *attB* sites) may be ‘flipped’ repeatedly into the *LR* orientation and back again by transient bursts of integrase and then integrase–RDF fusion recombinase.

The integrase–RDF fusion recombinase might be a useful platform for future work to enhance the efficiency and directionality of *attL* × *attR* recombination by protein engineering or directed evolution. It could also be used in genetic screens to identify the RDFs for integrases; candidate RDF reading frames could simply be cloned at the integrase C-terminus and the fusion protein activity could then be tested in *E. coli*. Similarly, it might be possible to find synthetic RDFs by cloning libraries of peptide-coding fragments at the integrase C-terminus and selecting for *attL* × *attR* recombination activity. We also envisage systems for conditional creation of fusion recombinases from integrase and RDF components by split-gene reassembly, or post-translationally by intein splicing technology.

## Supplementary Material

Supplementary Data
