# Supplementary material for: Control of serine integrase recombination directionality by fusion with the directionality factor
Source: Nucleic Acids Res. 2017 Jun 28;45(14):8635–45. doi: 10.1093/nar/gkx567 (PMC5737554; doi:10.1093/nar/gkx567)
Supplement: Supplementary Data [file gkx567_supp.pdf]

SUPPLEMENTARY FIGURE AND TABLE LEGENDS

Figure S1

Vectors used for construction of recombination reaction substrates. (A) pFM141 (the vector used to construct *in vivo* reaction substrates). Recombination (*att*) sites for ϕC31 integrase were introduced by replacing the stuffer sequence flanked by XbaI and NotI sites (Site A), and EcoRI and SacI sites (Site B) with synthetic double-stranded oligonucleotides (see Table S1). Bxb1 integrase substrates were made similarly from pMS183Δ, a variant of FM141 in which sites A and B are both flanked by EcoRI and SacI sites. (B) pFM122 (the vector used to construct *in vitro* reaction substrates). Recombination sites were cloned by replacement of the stuffer sequences between SpeI and NotI sites (Site A), and EcoRI and SacI sites (Site B) with double-stranded oligonucleotides containing *att* sites as shown in Table S1, flanked with the appropriate restriction sites.

Table S1

Sequences of the recombination (*att*) sites for ϕC31 integrase and Bxb1 integrase, used in this study. The central 2-bp overlap sequences of the *att* sites are highlighted in red font. The flanking restriction sites used in cloning double-stranded oligonucleotides into pFM141 and pFM122 (shown in Figure S1) are highlighted in blue; bases in italics are not in the synthetic oligonucleotides used for cloning the sites. Note that SpeI and XbaI-cut sites have compatible ends. The sequences as shown here give substrates with head-to-tail sites (i.e. recombination results in resolution/deletion). To make inversion substrates, the orientation of the sequence (black letters) in one of the double-stranded oligonucleotides (*attB* or *attL*) was reversed.

Table S2

Sequences of plasmids used in this study.

(1) Full sequence of pFEM33, the plasmid used for low-level expression of ϕC31.Int-gp3 fusion protein in *E. coli*. pFEM33 has a pMB1 origin of replication and an ampicillin-resistance gene. The ϕC31 integrase sequence is highlighted in cyan, and the gp3 sequence is highlighted in grey. The linker between these sequences is described in the main text (Figure 1C). The restriction sites referred to in the main text are highlighted in yellow: NdeI, CATATG; SpeI, ACTAGT; Acc65I, GGTACC; BglII, AGATCT; XhoI, CTCGAG. Stop codons (TAA) are in red font. The expression plasmid for ϕC31 integrase is similar, except that the gp3 sequence and the integrase-gp3 linker are

absent. The expression plasmid for gp3 is also similar except that the ϕC31 integrase and linker sequences are absent.

(2) The coding sequence for Bxb1 integrase, and (3) the coding sequence for gp47. Sequences of the expression plasmids for Bxb1 integrase, Bxb1.Int-gp47, and gp47 can be obtained by replacement of the relevant coding sequences in pFEM33.

(4) Full sequence of pFM141, the vector used to construct *in vivo* recombination substrates (see Figure S1). Restriction sites are highlighted in yellow: XbaI, TCTAGA; NotI, GCGGCCGC; EcoRI, GAATTC; SacI, GAGCTC. The region between the two *att* sites containing the *galK* gene (highlighted in cyan) is deleted or inverted by recombination, along with half of each *att* site.

(5) Full sequence of pFM122, the vector used to construct *in vitro* recombination substrates (see Figure S1). Restriction sites are highlighted in yellow: SpeI, ACTAGT; NotI, GCGGCCGC; EcoRI, GAATTC; SacI, GAGCTC. The region between the two *att* sites (highlighted in cyan) is deleted by recombination, along with half of each *att* site.

Figure S1

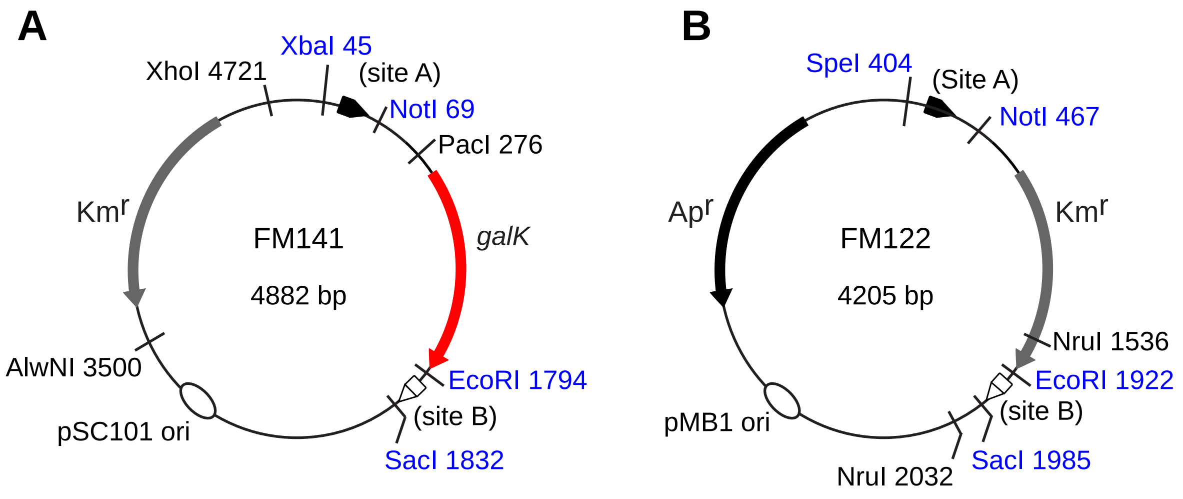
A
XhoI 4721
XbaI 45
(site A)
NotI 69
PacI 276
Km^r
FM141
4882 bp
galK
AlwNI 3500
EcoRI 1794
(site B)
pSC101 ori
SacI 1832
B
SpeI 404
(Site A)
NotI 467
Ap^r
FM122
4205 bp
Km^r
NruI 1536
EcoRI 1922
(site B)
pMB1 ori
SacI 1985
NruI 2032

Table S1

SacI … EcoRI

ϕC31 attB GAGCTCCCGCGGTGCGGGTGCCAGGGCGTGCCCTTGGGCTCCCCGGGCGCGTACTCCACCTGAATTC

CTCGAGGGCGCCACGCCCACGGTCCCGCACGGGAACCCGAGGGGCCCGCGCATGAGGTGGACTTAAG

NotI … SpeI

ϕC31 attP GCGGCCGC AGTAGTGCCCCAACTGGGGTAACCTTTGAGTTCTCTCAGTTGGGGGCGTAGGGACTAGT

CGCCGGCG TCATCACGGGGTTGACCCCATTGGAAACTCAAGAGAGTCAACCCCCGCATCCCTGATCA

SacI … EcoRI

ϕC31 attL GAGCTCCCGCGGTGCGGGTGCCAGGGCGTGCCCTTGAGTTCTCTCAGTTGGGGGCGTAGGGGAATTC

CTCGAGGGCGCCACGCCCACGGTCCCGCACGGGAACTCAAGAGAGTCAACCCCCGCATCCCCTTAAG

NotI … SpeI

ϕC31 attR GCGGCCGC AGTAGTGCCCCAACTGGGGTAACCTTTGGGCTCCCCGGGCGCGTACTCCACCTACTAGT

CGCCGGCG TCATCACGGGGTTGACCCCATTGGAAACCCGAGGGGCCCGCGCATGAGGTGGATGATCA

SacI … EcoRI

Bxb1 attB GAGCTCCCGCGGGCCGGCTTGTCGACGACGGGGGTCTCCGTCGTCAGGATCATCCGGTCCAGAATTC

CTCGAGGGCGCCCGGCCGAACAGCTGCTGCCGCCAGAGGCAGCAGTCCTAGTAGGCCAGGTCTTAAG

SacI … EcoRI

Bxb1 attP GAGCTCGTGGTTTGTCTGGTCAACCACCGCGGTCTCAGTGGTGTACGGTACAAACCCAGGAATTC

CTCGAGCACCAAACAGACCAGTTGGTGGCGCCAGAGTCACCACATGCCATGTTTGGGTCCTTAAG

SacI … EcoRI

Bxb1 attL GAGCTCCCGCGGGCCGGCTTGTCGACGACGGGGGTCTCAGTGGTGTACGGTACAAACCCAGGAATTC

CTCGAGGGCGCCCGGCCGAACAGCTGCTGCCGCCAGAGTCACCACATGCCATGTTTGGGTCCTTAAG

SacI … EcoRI

Bxb1 attR GAGCTCGTGGTTTGTCTGGTCAACCACCGCGGTCTCCGTCGTCAGGATCATCCGGTCCAGAATTC

CTCGAGCACCAAACAGACCAGTTGGTGGCGCCAGAGGCAGCAGTCCTAGTAGGCCAGGTCTTAAG

Table S2

(1) pFEM33 (ϕC31.Integrase-gp3 fusion *in vivo* expression plasmid) DNA sequence

GAATTCTAGAAATAATTTTGTTTAACTTTAAGAAGGAGATATACATATGGACACGTACGCGGGTGCTTACGACCGTCAGTCGCGCGAGCGCGAGAATTCGAGC
GCAGCAAGCCCAGCGACACAGCGTAGCGCCAACGAAGACAAGGCGGCCGACCTTCAGCGCGAAGTCGAGCGCGACGGGGGCCGGTTCAGGTTCGTCGGGCATT
TCAGCGAAGCGCCGGGCACGTCGGCGTTCGGGACGGCGGAGCGCCCGGAGTTCGAACGCATCCTGAACGAATGCCGCGCCGGGCGGCTCAACATGATCATTGT
CTATGACGTGTCGCGCTTCTCGCGCCTGAAGGTCATGGACGCGATTCCGATTGTCTCGGAATTGCTCGCCCTGGGCGTGACGATTGTTTCCACTCAGGAAGGC
GTCTTCCGGCAGGGAAACGTCATGGACCTGATTCACCTGATTATGCGGCTCGACGCGTCGCACAAAGAATCTTCGCTGAAGTCGGCGAAGATTCTCGACACGA
AGAACCTTCAGCGCGAATTGGGCGGGTACGTCGGCGGGAAGGCGCCTTACGGCTTCGAGCTTGTTTCGGAGACGAAGGAGATCACGCGCAACGGCCGAATGGT
CAATGTCGTCATCAACAAGCTTGCGCACTCGACCACTCCCCTTACCGGACCCTTCGAGTTCGAGCCCGACGTAATCCGGTGGTGGTGGCGTGAGATCAAGACG
CACAAACACCTTCCCTTCAAGCCGGGCAGTCAAGCCGCCATTCACCCGGGCAGCATCACGGGGCTTTGTAAGCGCATGGACGCTGACGCCGTGCCGACCCGGG
GCGAGACGATTGGGAAGAAGACCGCTTCAAGCGCCTGGGACCCGGCAACCGTTATGCGAATCCTTCGGGACCCGCGTATTGCGGGCTTCGCCGCTGAGGTGAT
CTACAAGAAGAAGCCGGACGGCACGCCGACCACGAAGATTGAGGGTTACCGCATTCAGCGCGACCCGATCACGCTCCGGCCGGTCGAGCTTGATTGCGGACCG
ATCATCGAGCCCGCTGAGTGGTATGAGCTTCAGGCGTGGTTGGACGGCAGGGGGCGCGGCAAGGGGCTTTCCCGGGGGCAAGCCATTCTGTCCGCCATGGACA
AGCTGTACTGCGAGTGTGGCGCCGTCATGACTTCGAAGCGCGGGGAAGAATCGATCAAGGACTCTTACCGCTGCCGTCGCCGGAAGGTGGTCGACCCGTCCGC
ACCTGGGCAGCACGAAGGCACGTGCAACGTCAGCATGGCGGCACTCGACAAGTTCGTTGCGGAACGCATCTTCAACAAGATCAGGCACGCCGAAGGCGACGAA
GAGACGTTGGCGCTTCTGTGGGAAGCCGCCCGACGCTTCGGCAAGCTCACTGAGGCGCCTGAGAAGAGCGGCGAACGGGCGAACCTTGTTGCGGAGCGCGCCG
ACGCCCTGAACGCCCTTGAAGAGCTGTACGAAGACCGCGCGGCAGGCGCGTACGACGGACCCGTTGGCAGGAAGCACTTCCGGAAGCAACAGGCAGCGCTGAC
GCTCCGGCAGCAAGGGGCGGAAGAGCGGCTTGCCGAACTTGAAGCCGCCGAAGCCCCGAAGCTTCCCCTTGACCAATGGTTCCCCGAAGACGCCGACGCTGAC
CCGACCGGCCCTAAGTCGTGGTGGGGGCGCGCGTCAGTAGACGACAAGCGCGTGTTCGTCGGGCTCTTCGTAGACAAGATCGTTGTCACGAAGTCGACTACGG
GCAGGGGGCAGGGAACGCCCATCGAGAAGCGCGCTTCGATCACGTGGGCGAAGCCGCCGACCGACGACGACGAAGACGACGCCCAGGACGGCACGGAAGACGT
AGCGGCGACTAGTAGATCTGGTACCGCGAAGCGTTCGATCTGGGCCGGAGACGAAGACAACAAGCCGAAGAAGCGTGAGACGTACGCCGACGACACCGTTGGG
CGTTTCCACTCCGGCTACAGCGAGACGAATGAGCGGGGCAAGGTCGTTCCCGTCGCGCTCGACAAGTGGCGTATCTCGACCGGCGAACAGTCCGTTGCGGACG
CTGTGGCTCAGCTCTTCGGCGGCACCCCGGTTGAGAACGAAGAGAGCACGTCTGAGAACTTCATCGACGTGTTCACCGACCGGCCGAAGGTCCCCGTGATCAT
TGAGGCTGACGGTATCCACTGGGATATGAAGCTTTGGCTGAACGGCAAGCTGAAGCACCACTGCGACGGCTTTGACTTCGTGTCGCACGCCGACGAAGAGATG
ATCGGTCAGCCGTGCGGGTGCCCGAAGCTCTTCGATGAGCGGAAGGCAGCGGCGAAGGAGTACGACGCGCCGAACCCGGCAATCACCGTGACCTTCACGCTCG
CCGACGACCCGGAGCTTGGCCGCTTCAAGTTCCAGACCGGTTCTTGGACGCTCTTCAAGGTTCTGCACGAAGCCGAAGACGACGTTGAGCGCGTCGGCAAGGG
TGGCGCTGTGCTCGCCAATCTCGAACTTGAGCTTGTGGAGTACACGCCGAAGCGTGGCCCGATGCGGAACAAGCTTGTGAGCTACTACAAGCCGACCATCACG
GTTCTGAAGTCGTACAACGACGCGATTGCCGACTAATAACTCGAGTGTACCTCTAGAGCTTGAGTATTCTATAGTGTCACCTAAATAGCTTGGCGTAATCATG
GTCATAGCTGTTTCCTGTGTGAAATTGTTATCCGCTCACAATTCCACACAACATACGAGCCGGAAGCATAAAGTGTAAAGCCTGGGGTGCCTAATGAGTGAGC
TAACTCACATTAATTGCGTTGCGCTCACTGCCCGCTTTCCAGTCGGGAAACCTGTCGTGCCAGCTGCATTAATGAATCGGCCAACGCGCGGGGAGAGGCGGTT
TGCGTATTGGGCGCTCTTCCGCTTCCTCGCTCACTGACTCGCTGCGCTCGGTCGTTCGGCTGCGGCGAGCGGTATCAGCTCACTCAAAGGCGGTAATACGGTT
ATCCACAGAATCAGGGGATAACGCAGGAAAGAACATGAATTAATTCTCATGTTTGACAGCTTATCATaGATTAGCTTTAATGCGGTAGTTTATCACAGTTAAA
TTGCTAACGCAGTCAGGCACCGTGTATGAAATCTAACAATGCGCTCATCGTCATCCTCGGCACCGTCACCCTGGATGCTGTAGGCATAGGCTTGGTTATGCCG
GTACTGCCGGGCCTCTTGCGGGATCGACGCGAGGCTGGATGGCCTTCCCCATTATGATTCTTCTCGCTTCCGGCGGCATCGGGATGCCCGCGTTGCAGGCCAT
GCTGTCCAGGCAGGTAGATGACGACCATCAGGGACAGCTTCAAGGATCGCTCGCGGCTCTTACCAGCCTAACTTCGATCACTGGACCGCTGATCGTCACGGCG
ATTTATGCCGCCTCGGCGAGCACATGGAACGGGTTGGCATGGATTGTAGGCGCCGCCCTATACCTTGTCTGCCTCCCCGCGTTGCGTCGCGGTGCATGGAGCC
GGGCCACCTCGACCTGAATGGAAGCCGGCGGCACCTCGCTAACGGATTCACCACTCCAAGAATTGGAGCCAATCAATTCTTGCGGAGAACTGTGAATGCGCAA
ACCAACCCTTGGCAGAACATATCCATCGCGTCCGCCATCTCCAGCAGCCGCACGCGGCGCATCTCGGGCAGCGTTGGGTCCTGGCCACGGGTGCGCATGATCG
TGCTCCTGTCGTTGAGGACCCGGCTAGGCTGGCGGGGTTGCCTTACTGGTTAGCAGAATGAATCACCGATACGCGAGCGAACGTGAAGCGACTGCTGCTGCAA
AACGTCTGCGACCTGAGCAACAACATGAATGGTCTTCGGTTTCCGTGTTTCGTAAAGTCTGGAAACGCGGAAGTCAGCGCCCTGCACCATTATGTTCCGGATC
TGCATCGCAGGATGCTGCTGGCTACCCTGTGGAACACCTACATCTGTATTAACGAAGCGCTGGCATTGACCCTGAGTGATTTTTCTCTGGTCCCGCCGCATCC
ATACCGCCAGTTGTTTACCCTCACAACGTTCCAGTAACCGGGCATGTTCATCATCAGTAACCCGTATCGTGAGCATCCTCTCTCGTTTCATCGGTATCATTAC
CCCCATGAACAGAAATTCCCCCTTACACGGAGGCATCAAGTGACCAAACAGGAAAAAACCGCCCTTAACATGGCCCGCTTTATCAGAAGCCAGACATTAACGC
TTCTGGAGAAACTCAACGAGCTGGACGCGGATGAACAGGCAGACATCTGTGAATCGCTTCACGACCACGCTGATGAGCTTTACCGCAGCTGCCTCGCGCGTTT
CGGTGATGACGGTGAAAACCTCTGACACATGCAGCTCCCGGAGACGGTCACAGCTTGTCTGTAAGCGGATGCCGGGAGCAGACAAGCCCGTCAGGGCGCGTCA
GCGGGTGTTGGCGGGTGTCGGGGCGCAGCCATGACCCAGTCACGTAGCGATAGCGGAGTGTATACTGGCTTAACTATGCGGCATCAGAGCAGATTGTACTGAG
AGTGCACCATatATGCGGTGTGAAATACCGCACAGATGCGTAAGGAGAAAATACCGCATCAGGCGCTCTTCCGCTTCCTCGCTCACTGACTCGCTGCGCTCGG
TCGTTCGGCTGCGGCGAGCGGTATCAGCTCACTCAAAGGCGGTAATACGGTTATCCACAGAATCAGGGGATAACGCAGGAAAGAACATGTGAGCAAAAGGCCA
GCAAAAGGCCAGGAACCGTAAAAAGGCCGCGTTGCTGGCGTTTTTCCATAGGCTCCGCCCCCCTGACGAGCATCACAAAAATCGACGCTCAAGTCAGAGGTGG
CGAAACCCGACAGGACTATAAAGATACCAGGCGTTTCCCCCTGGAAGCTCCCTCGTGCGCTCTCCTGTTCCGACCCTGCCGCTTACCGGATACCTGTCCGCCT
TTCTCCCTTCGGGAAGCGTGGCGCTTTCTCATAGCTCACGCTGTAGGTATCTCAGTTCGGTGTAGGTCGTTCGCTCCAAGCTGGGCTGTGTGCACGAACCCCC
CGTTCAGCCCGACCGCTGCGCCTTATCCGGTAACTATCGTCTTGAGTCCAACCCGGTAAGACACGACTTATCGCCACTGGCAGCAGCCACTGGTAACAGGATT
AGCAGAGCGAGGTATGTAGGCGGTGCTACAGAGTTCTTGAAGTGGTGGCCTAACTACGGCTACACTAGAAGGACAGTATTTGGTATCTGCGCTCTGCTGAAGC
CAGTTACCTTCGGAAAAAGAGTTGGTAGCTCTTGATCCGGCAAACAAACCACCGCTGGTAGCGGTGGTTTTTTTGTTTGCAAGCAGCAGATTACGCGCAGAAA
AAAAGGATCTCAAGAAGATCCTTTGATCTTTTCTACGGGGTCTGACGCTCAGTGGAACGAAAACTCACGTTAAGGGATTTTGGTCATGAGATTATCAAAAAGG
ATCTTCACCTAGATCCTTTTAAATTAAAAATGAAGTTTTAAATCAATCTAAAGTATATATGAGTAAACTTTGGCTGACAGTTACCAATGCTTAATCAGTGAGG
CACCTATCTCAGCGATCTGTCTATTTCGTTCATCCATAGTTGCCTGACTCCCCGTCGTGTAGATAACTACGATACGGGAGGGCTTACCATCTGGCCCCAGTGC
TGCAATGATACCGCGAGACCCACGCTCACCGGCTCCAGATTTATCAGCAATAAACCAGCCAGCCGGAAGGGCCGAGCGCAGAAGTGGTCCTGCAACTTTATCC
GCCTCCATCCAGTCTATTAATTGTTGCCGGGAAGCTAGAGTAAGTAGTTCGCCAGTTAATAGTTTGCGCAACGTTGTTGCCATTGCtgcaGGCATCGTGGTGT
CACGCTCGTCGTTTGGTATGGCTTCATTCAGCTCCGGTTCCCAACGATCAAGGCGAGTTACATGATCCCCCATGTTGTGCAAAAAAGCGGTTAGCTCCTTCGG
TCCTCCGATCGTTGTCAGAAGTAAGTTGGCCGCAGTGTTATCACTCATGGTTATGGCAGCACTGCATAATTCTCTTACTGTCATGCCATCCGTAAGATGCTTT
TCTGTGACTGGTGAGTACTCAACCAAGTCATTCTGAGAATAGTGTATGCGGCGACCGAGTTGCTCTTGCCCGGCGTCAACACGGGATAATACCGCGCCACATA
GCAGAACTTTAAAAGTGCTCATCATTGGAAAACGTTCTTCGGGGCGAAAACTCTCAAGGATCTTACCGCTGTTGAGATCCAGTTCGATGTAACCCACTCGTGC
ACCCAACTGATCTTCAGCATCTTTTACTTTCACCAGCGTTTCTGGGTGAGCAAAAACAGGAAGGCAAAATGCCGCAAAAAAGGGAATAAGGGCGACACGGAAA
TGTTGAATACTCATACTCTTCCTTTTTCAATATTATTGAAGCATTTATCAGGGTTATTGTCTCATGAGCGGATACATATTTGAATGTATTTAGAAAAATAAAC
AAATAGGGGTTCCGCGCACATTTCCCCGAAAAGTGCCACCTGACGTCTAAGAAACCATTATTATCATGACATTAACCTATAAAAATAGGCGTATCACGAGGCC
CTTTCGTCTCGCGCGTTTCGGTGATGACGGTGAAAACCTCTGACACATGCAGCTCCCGGAGACGGTCACAGCTTGTCTGTAAGCGGATGCCGGGAGCAGACAA
GCCCGTCAGGGCGCGTCAGCGGGTGTTGGCGGGTGTCGGGGCTGGCTTAACTATGCGGCATCAGAGCAGATTGTACTGAGAGTGCACCATatATGCGGTGTGA
AATACCGCACAGATGCGTAAGGAGAAAATACCGCATCAGGCGAAATTGTAAACGTTAATATTTTGTTAAAATTCGCGTTAAATATTTGTTAAATCAGCTCATT
TTTTAACCAATAGGCCGAAATCGGCAAAATCCCTTATAAATCAAAAGAATAGACCGAGATAGGGTTGAGTGTTGTTCCAGTTTGGAACAAGAGTCCACTATTA
AAGAACGTGGACTCCAACGTCAAAGGGCGAAAAACCGTCTATCAGGGCGATGGCCCACTACGTGAACCATCACCCAAATCAAGTTTTTTGCGGTCGAGGTGCC
GTAAAGCTCTAAATCGGAACCCTAAAGGGAGCCCCCGATTTAGAGCTTGACGGGGAAAGCCGGCGAACGTGGCGAGAAAGGAAGGGAAGAAAGCGAAAGGAGC
GGGCGCTAGGGCGCTGGCAAGTGTAGCGGTCACGCTGCGCGTAACCACCACACCCGCCGCGCTTAATGCGCCGCTACAGGGCGCGTCCATTCGCCATTCAGGC
TGCGCAACTGTTGGGAAGGGCGATCGGTGCGGGCCTCTTCGCTATTACGCCAGCTGGCGAAAGGGGGATGTGCTGCAAGGCGATTAAGTTGGGTAACGCCAGG
GTTTTCCCAGTCACGACGTTGTAAAACGACGGCCAGTGAATTGTAATACGACTCACTATAGGGC

(2) Bxb1 integrase ORF sequence

```
ATGCGTGCACTGGTTGTTATTCGTCTGAGCCGTGTTACCGATGCAACCACCAGTCCGGAACGTCAGCTGGAAAGCTGTCAGCAGCTGTGTGCACAGCGTGGTT
GGGATGTTGTTGGTGTTGCCGAAGATCTGGATGTTAGCGGTGCAGTTGATCCGTTTGATCGTAAACGTCGTCCGAATCTGGCACGTTGGCTGGCATTTGAAGA
ACAGCCGTTTGATGTTATTGTTGCCTATCGTGTTGATCGTCTGACCCGTAGCATTCGTCATCTGCAGCAGCTGGTTCATTGGGCAGAAGATCATAAAAAACTG
GTTGTGAGCGCAACCGAAGCACATTTTGATACCACCACCCCGTTTGCAGCAGTTGTTATTGCACTGATGGGCACCGTTGCACAGATGGAACTGGAAGCAATTA
AAGAACGTAATCGTAGCGCAGCCCATTTTAACATTCGTGCAGGTAAATATCGTGGTAGCCTGCCTCCGTGGGGTTATCTGCCGACCCGTGTTGATGGTGAATG
GCGTCTGGTTCCGGATCCTGTTCAGCGTGAACGTATTCTGGAAGTTTATCATCGTGTGGTGGATAATCATGAACCGCTGCATCTGGTTGCACATGATCTGAAT
CGTCGTGGTGTTCTGAGCCCGAAAGATTATTTTGCACAGCTGCAGGGTCGTGAACCGCAGGGTCGCGAATGGTCAGCAACCGCACTGAAACGTAGCATGATTA
GCGAAGCAATGCTGGGTTATGCAACCCTGAATGGTAAAACCGTTCGTGATGATGATGGTGCACCGCTGGTTCGTGCAGAACCGATTCTGACACGTGAACAGCT
GGAAGCACTGCGTGCCGAACTGGTTAAAACCAGCCGTGCAAAACCGGCAGTTAGCACCCCGAGCCTGCTGCTGCGTGTTCTGTTTTGTGCAGTTTGTGGTGAA
CCGGCATACAAATTTGCCGGTGGTGGTCGTAAACATCCGCGTTATCGTTGTCGTAGCATGGGTTTTCCGAAACATTGTGGTAATGGTACAGTTGCAATGGCAG
AATGGGATGCATTTTGCGAAGAACAGGTTCTGGATCTGCTGGGTGATGCCGAACGTCTGGAAAAAGTTTGGGTTGCAGGTAGCGATAGCGCAGTTGAACTGGC
CGAAGTTAATGCAGAACTGGTTGATCTGACCAGCCTGATTGGTAGTCCGGCATATCGTGCCGGTAGTCCGCAGCGTGAAGCACTGGATGCACGTATTGCAGCA
CTGGCAGCACGTCAAGAAGAACTGGAAGGTCTGGAAGCACGTCCGAGCGGTTGGGAATGGCGTGAAACCGGTCAGCGTTTTGGTGATTGGTGGCGTGAGCAGG
ATACCGCAGCAAAAAATACCTGGCTGCGTAGTATGAATGTTCGCCTGACCTTTGATGTTCGCGGTGGCCTGACCCGCACCATTGATTTTGGCGATCTGCAAGA
ATATGAACAGCATCTGCGTCTGGGTAGCGTTGTTGAACGTCTGCATACCGGCATGAGC[STOP]
```

(3) gp47 ORF sequence

```
ATGCATCATCATCACCATCATACCAGCATGACCCAGCGTATTGTTTTTCTGCCGGATACCCAGCTGCCGTTTGAAGCACGTAAAGAAATGCAGGCAGTGATTC
GTTTTATTGGTGATGTTCAGCCGTATGGTGTTGTTCATATCGGTGATGTGCTGGATCTGCCGCAGCCGAGCCGTTGGAATCGTGGCACCAAAGGTGAATTTGA
AGGTAGCGTTTATCGTGATGCAGACTACGCCAAAAAAAACCTGATGGAACCGCTGCGTAAAGTTTATGATGGTTGGATTGGTATGCATGAAGGCAATCATGAT
CTGCGTGCACGTGAATATCTGGCAAAAAATGCACCGGCACTGGAAGGCACCCATGCATTTGATATTGATGTTCTGCTGGATTTTGATGGCTTTGGTGTTGAAC
TGCTGCCGGATTTTTATGATATTGCACCGGGTTGGATTAGCACCCATGGTCACATGGGTAAAATGACCCTGAGCCAGATTGCAGGTAGCACCGCACTGAATGG
TGCCAAAAAATTCGGTAAAAGCGTTGTTTGTGGTCATACCCATCGTCAGGCAGTTGTTAGCCATAGCTTTGGTTATGGTGGTAGCGTTCGTAAAACCGTTACC
GGTATGGAAGTTGGTCATCTGATGGATATGAAAAAAGCCAACTATCTGAAAGGTGGTGCAGGTAATTGGCAGATGGGTTTTGGTATGCTGACCGTTGATGGTA
AACATGTGAAAGCAGAAATTGTTCCGATTCTGGGTGGCAAATTTACCGTGGATGGTCAGGTTTGGGAAGTG[STOP]
```

(4) pFM141 (*in vivo* recombination cloning plasmid) DNA sequence

```
GCATCGTGGTGTCACGCTCGGCAtggccggcGCTAGCAAGATCTTCTAGAAAACGGCCGTTGTACATTGCGGCCGCGCATGCATCGATAGATCCAATTGCCGT
GACGCAGGCATGTTTCTCAATAACGAAATTTGATAAAATCCCGCTCTTTCATAACATTATTTCAGCCTTCTTCAGGGCTGACTGTTTGCATAAAAATTCATCT
GTATGCACAATAATGTTGTATCAACCACCATATCGGGTGACTTATGCGAAGCTCGGCTAAGCAAGAAGATTAATTAATAAAGCATTTGGGCGAACGCGAATTA
ATTCACTGGCCGTCGTTTTACAACGTCGTGACTGGGAAAACCCTGGCGTTACCCAACTTAATCGCCTTGCAGCACATCCCCCTTTCGCCAGGGGCAATAAGGG
CTGCACGCGCACTTTTATCCGCCTCTGCTGCGCTCCGCCACCGTACGTAAATTTATGGTTGGTTATGAAATGCTGGCAGAGACCCAGCGAGACCTGACCGCAG
AACAGGCAGCAGAGCGTTTGCGCGCAGTCAGCGATATCCATTTTCGCGAATCCGGAGTGTAAGAAATGAGTCTGAAAGAAAAAACACAATCTCTGTTTGCCAA
CGCATTTGGCTACCCTGCCACTCACACCATTCAGGCGCCTGGCCGCGTGAATTTGATTGGTGAACACACCGACTACAACGACGGTTTCGTTCTGCCCTGCGCG
ATTGATTATCAAACCGTGATCAGTTGTGCACCACGCGATGACCGTAAAGTTCGCGTGATGGCAGCCGATTATGAAAATCAGCTCGACGAGTTTTCCCTCGATG
CGCCCATTGTCGCACATGAAAACTATCAATGGGCTAACTACGTTCGTGGCGTGGTGAAACATCTGCAACTGCGTAACAACAGCTTCGGCGGCGTGGACATGGT
GATCAGCGGCAATGTGCCGACGGGTGCCGGGTTAAGTTCTTCCGCTTCACTGGAAGTCGCGGTCGGAACCGTATTGCAGCAGCTTTATCATCTGCCGCTGGAC
GGCGCACAAATCGCGCTTAACGGTCAGGAAGCAGAAAACCAGTTTGTAGGCTGTAACTGCGGGATCATGGATCAGCTAATTTCCGCGCTCGGCAAGAAAGATC
ATGCCTTGCTGATCGATTGCCGCTCACTGGGGACCAAAGCAGTTTCCATGCCCAAAGGTGTGGCTGTCGTCATCATCAACAGTAACTTCAAACGTACCCTGGT
TGGCAGCGAATACAACACCCGTCGTGAACAGTGCGAAACCGGTGCGCGTTTCTTCCAGCAGCCAGCCCTGCGTGATGTCACCATTGAAGAGTTCAACGCTGTT
GCGCATGAACTGGACCCGATCGTGGCAAAACGCGTGCGTCATATACTGACTGAAAACGCCCGCACCGTTGAAGCTGCCAGCGCGCTGGAGCAAGGCGACCTGA
AACGTATGGGCGAGTTGATGGCGGAGTCTCATGCCTCTATGCGCGATGATTTCGAAATCACCGTGCCGCAAATTGACACTCTGGTAGAAATCGTCAAAGCTGT
GATTGGCGACAAAGGTGGCGTACGCATGACCGGCGGCGGATTTGGCGGCTGTATCGTCGCGCTGATCCCGGAAGAGCTGGTGCCTGCCGCACAGCAAGCTGTC
GCTGAACAATATGAAGCAAAAACAGGTATTAAAGAGACTTTTTACGTTTGTAAACCATCACAAGGAGCAGGACAGTGCTGAACGAAACTCCCGCACTGCAGGA
TCgatcCATATGACGTCGACGCGTCTGCAGAAGCTTCTAGGTGAATTCGACATCAGGTTAGGGTATGCCTCAACCTGACGGAGCTCCCGGGTACCAtggCGGT
GAACAGTTGTTCTACTTTTGTTTGTTAGTCTTGATGCTTCACTGATAGATACAAGAGCCATAAGAACCTCAGATCCTTCCGTATTTAGCCAGTATGTTCTCTA
GTGTGGTTCGTTGTTTTTGCGTGAGCCATGAGAACGAACCATTGAGATCATGCTTACTTTGCATGTCACTCAAAAATTTTGCCTCAAAACTGGTGAGCTGAAT
TTTTGCAGTTAAAGCATCGTGTAGTGTTTTTCTTAGTCCGTTACGTAGGTAGGAATCTGATGTAATGGTTGTTGGTATTTTGTCACCATTCATTTTTATCTGG
TTGTTCTCAAGTTCGGTTACGAGATCCATTTGTCTATCTAGTTCAACTTGGAAAATCAACGTATCAGTCGGGCGGCCTCGCTTATCAACCACCAATTTCATAT
TGCTGTAAGTGTTTAAATCTTTACTTATTGGTTTCAAAACCCATTGGTTAAGCCTTTTAAACTCATGGTAGTTATTTTCAAGCATTAACATGAACTTAAATTC
ATCAAGGCTAATCTCTATATTTGCCTTGTGAGTTTTCTTTTGTGTTAGTTCTTTTAATAACCACTCATAAATCCTCATAGAGTATTTGTTTTCAAAAGACTTA
ACATGTTCCAGATTATATTTTATGAATTTTTTTAACTGGAAAAGATAAGGCAATATCTCTTCACTAAAAACTAATTCTAATTTTTCGCTTGAGAACTTGGCAT
AGTTTGTCCACTGGAAAATCTCAAAGCCTTTAACCAAAGGATTCCTGATTTCCACAGTTCTCGTCATCAGCTCTCTGGTTGCTTTAGCTAATACACCATAAGC
ATTTTCCCTACTGATGTTCATCATCTGAGCGTATTGGTTATAAGTGAACGATACCGTCCGTTCTTTCCTTGTAGGGTTTTCAATCGTGGGGTTGAGTAGTGCC
ACACAGCATAAAATTAGCTTGGTTTCATGCTCCGTTAAGTCATAGCGACTAATCGCTAGTTCATTTGCTTTGAAAACAACTAATTCAGACATACATCTCAATT
GGTCTAGGTGATTTTAATCACTATACCAATTGAGATGGGCTAGTCAATGATAATTACTAGTCCTTTTCCTTTGAGTTGTGGGTATCTGTAAATTCTGCTAGAC
CTTTGCTGGAAAACTTGTAAATTCTGCTAGACCCTCTGTAAATTCCGCTAGACCTTTGTGTGTTTTTTTTGTTTATATTCAAGTGGTTATAATTTATAGAATA
AAGAAAGAATAAAAAAAGATAAAAAGAATAGATCCCAGCCCTGTGTATAACTCACTACTTTAGTCAGTTCCGCAGTATTACAAAAGGATGTCGCAAACGCTGT
TTGCTCCTCTACAAAACAGACCTTAAAACCCTAAAGGCTTAAGTAGCACCCTCGCAAGCTCGGGCAAATCGCTGAATATTCCTTTTGTCTCCGACCATCAGGC
ACCTGAGTCGCTGTCTTTTTCGTGACATTCAGTTCGCTGCGCTCACGGCTCTGGCAGTGAATGGGGGTAAATGGCACTACAGGCGCCTTTTATGGATTCATGC
AAGGAAACTACCCATAATACAAGAAAAGCCCGTCACGGGCTTCTCAGGGCGTTTTATGGCGGGTCTGCTATGTGGTGCTATCTGACTTTTTGCTGTTCAGCAG
TTCCTGCCCTCTGATTTTCCAGTCTGACCACTTCGGATTATCCCGTGACAGGTCATTCAGACTGGCTAATGCACCCAGTAAGGCAGCGGTATCATCAACAGGC
TTACCCGTCTTACTGTCCCGGATCCGTCGACCTGCAGGGGGGGGGGGGGCGCTGAGGTCTGCCTCGTGAAGAAGGTGTTGCTGACTCATACCAGGCCTGAATCG
CCCCATCATCCAGCCAGAAAGTGAGGGAGCCACGGTTGATGAGAGCTTTGTTGTAGGTGGACCAGTTGGTGATTTTGAACTTTTGCTTTGCCACGGAACGGTC
TGCGTTGTCGGGAAGATGCGTGATCTGATCCTTCAACTCAGCAAAAGTTCGATTTATTCAACAAAGCCGCCGTCCCGTCAAGTCAGCGTAATGCTCTGCCAGT
GTTACAACCAATTAACCAATTCTGATTAGAAAAACTCATCGAGCATCAAATGAAACTGCAATTTATTCATATCAGGATTATCAATACCATATTTTTGAAAAAG
CCGTTTCTGTAATGAAGGAGAAAACTCACCGAGGCAGTTCCATAGGATGGCAAGATCCTGGTATCGGTCTGCGATTCCGACTCGTCCAACATCAATACAACCT
ATTAATTTCCCCTCGTCAAAAATAAGGTTATCAAGTGAGAAATCACCATGAGTGACGACTGAATCCGGTGAGAATGGCAAAAGCTTATGCATTTCTTTCCAGA
CTTGTTCAACAGGCCAGCCATTACGCTCGTCATCAAAATCACTCGCATCAACCAAACCGTTATTCATTCGTGATTGCGCCTGAGCGAGACGAAATACGCGATC
GCTGTTAAAAGGACAATTACAAACAGGAATCGAATGCAACCGGCGCAGGAACACTGCCAGCGCATCAACAATATTTTCACCTGAATCAGGATATTCTTCTAAT
ACCTGGAATGCTGTTTTCCCGGGGATCGCAGTGGTGAGTAACCATGCATCATCAGGAGTACGGATAAAATGCTTGATGGTCGGAAGAGGCATAAATTCCGTCA
GCCAGTTTAGTCTGACCATCTCATCTGTAACATCATTGGCAACGCTACCTTTGCCATGTTTCAGAAACAACTCTGGCGCATCGGGCTTCCCATACAATCGATA
GATTGTCGCACCTGATTGCCCGACATTATCGCGAGCCCATTTATACCCATATAAATCAGCATCCATGTTGGAATTTAATCGCGGCCTCGAGCAAGACGTTTCC
CGTTGAATATGGCTCATAACACCCCTTGTATTACTGTTTATGTAAGCAGACAGTTTATTGTTCATGATGATATATTTTTATCTTGTGCAATGTAACATCAGA
GATTTTGAGACACAACGTGGCTTTCCCCCCCCCCCCCTGCAG
```

(5) pFM122 (*in vitro* recombination cloning plasmid) DNA sequence

ACTGCCGGGCCTCTTGCGGGATATCGTCCATTCCGACAGCATCGCCAGTCACTATGGCGTGCTGCTAGCGCCATTCGCCATTCAGGCTACGCAACTGTTGGGA
AGGGCGATCGGTGCGGGCCTCTTCGCTATTACGCCAGCTGGCGAAGGGGGGATGTGCTGCAAGGCGATTAAGTTGGGTAACGCCAGGGTTTTCCCAGTCACGA
CGTTGTAAAACGACGGCCAGTGAATTGCCGGCGATATCGGATCCATATGACGTCGACGCGTCTGCAGAAGCTTCTAGAATGTACCTTAAATCGAATATCAGAC
ACGATGTGTCTATTATGCCAAAATGACGATTTAATGGACACTCGAGCGAAGCCGAAtttccggattatgtatacCAATTGCTTAAGCCTAGGCGACTAGTTAA
TTTTAGTTTCTCGTTTCTTCTTCTTCCAACGAGAGAAAACGAGGAACTAAACAAGCGGCCGCGGGTACCATGGCATGCATCGATAGATCCGTCGACCTGCAGG
GGGGGGGGGGCGCTGAGGTCTGCCTCGTGAAGAAGGTGTTGCTGACTCATACCAGGCCTGAATCGCCCCATCATCCAGCCAGAAAGTGAGGGAGCCACGGTTG
ATGAGAGCTTTGTTGTAGGTGGACCAGTTGGTGATTTTGAACTTTTGCTTTGCCACGGAACGGTCTGCGTTGTCGGGAAGATGCGTGATCTGATCCTTCAACT
CAGCAAAAGTTCGATTTATTCAACAAAGCCGCCGTCCCGTCAAGTCAGCGTAATGCTCTGCCAGTGTTACAACCAATTAACCAATTCTGATTAGAAAAACTCA
TCGAGCATCAAATGAAACTGCAATTTATTCATATCAGGATTATCAATACCATATTTTTGAAAAAGCCGTTTCTGTAATGAAGGAGAAAACTCACCGAGGCAGT
TCCATAGGATGGCAAGATCCTGGTATCGGTCTGCGATTCCGACTCGTCCAACATCAATACAACCTATTAATTTCCCCTCGTCAAAAATAAGGTTATCAAGTGA
GAAATCACCATGAGTGACGACTGAATCCGGTGAGAATGGCAAAAGCTTATGCATTTCTTTCCAGACTTGTTCAACAGGCCAGCCATTACGCTCGTCATCAAAA
TCACTCGCATCAACCAAACCGTTATTCATTCGTGATTGCGCCTGAGCGAGACGAAATACGCGATCGCTGTTAAAAGGACAATTACAAACAGGAATCGAATGCA
ACCGGCGCAGGAACACTGCCAGCGCATCAACAATATTTTCACCTGAATCAGGATATTCTTCTAATACCTGGAATGCTGTTTTCCCGGGGATCGCAGTGGTGAG
TAACCATGCATCATCAGGAGTACGGATAAAATGCTTGATGGTCGGAAGAGGCATAAATTCCGTCAGCCAGTTTAGTCTGACCATCTCATCTGTAACATCATTG
GCAACGCTACCTTTGCCATGTTTCAGAAACAACTCTGGCGCATCGGGCTTCCCATACAATCGATAGATTGTCGCACCTGATTGCCCGACATTATCGCGAGCCC
ATTTATACCCATATAAATCAGCATCCATGTTGGAATTTAATCGCGGCCTCGAGCAAGACGTTTCCCGTTGAATATGGCTCATAACACCCCTTGTATTACTGTT
TATGTAAGCAGACAGTTTTATTGTTCATGATGATATATTTTTATCTTGTGCAATGTAACATCAGAGATTTTGAGACACAACGTGGCTTTCCCCCCCCCCCCTG
CAGGTCGACGGATCCATATGACGTCGACGCGTCTGCAGAAGCTTCTAGAATGTACCTTAAATCGAATATCAGACACGATGTGTCTATTATGCCAAAATGACGA
TTTAATGGACACTCGAGCGAAGCCGAAtttccggattatgtataccaattgtattgttaacaccggtGAATTCAATTAAGTGTTTAGTTCCCTCTTTGCGTCC
TTCATAGCTTGATCCGAAAAAGTTACAGAGCTCGCCGGGTACCATGGCATGCATCGATAGATCTCgatcGAGGCCTCGCGAGCTTGGCGTAATCATGGTCATA
GCTGTTTCCTGTGTGAAATTGTTATCCGCTCACAATTCCACACAACATACGAGCCGGAAGCATAAAGTGTAAAGCCTGGGGTGCCTAATGAGTGAGGTAACTC
ACATTAATTGCGTTGCGCTCACTGCCCGCTTTCCAGTCGGGAAACCTGTCGTGCCAGCTGCATTAATGAATCGGCCAACGCGCGGGGAGAGGCGGTTTGCGTA
TTGGCGCTCTTCCGCTTCCTCGCTCACTGACTCGCTGCGCTCGGTCGTTCGGCTGCGGCGAGCGGTATCAGCTCACTCAAAGGCGGTAATACGGTTATCCACA
GAATCAGGGGATAACGCAGGAAAGAACATGTGAGCAAAAGGCCAGCAAAAGGCCAGGAACCGTAAAAAGGCCGCGTTGCTGGCGTTTTTCCATAGGCTCCGCC
CCCCTGACGAGCATCACAAAAATCGACGCTCAAGTCAGAGGTGGCGAAACCCGACAGGACTATAAAGATACCAGGCGTTTCCCCCTGGAAGCTCCCTCGTGCG
CTCTCCTGTTCCGACCCTGCCGCTTACCGGATACCTGTCCGCCTTTCTCCCTTCGGGAAGCGTGGCGCTTTCTCAATGCTCACGCTGTAGGTATCTCAGTTCG
GTGTAGGTCGTTCGCTCCAAGCTGGGCTGTGTGCACGAACCCCCCGTTCAGCCCGACCGCTGCGCCTTATCCGGTAACTATCGTCTTGAGTCCAACCCGGTAA
GACACGACTTATCGCCACTGGCAGCAGCCACTGGTAACAGGATTAGCAGAGCGAGGTATGTAGGCGGTGCTACAGAGTTCTTGAAGTGGTGGCCTAACTACGG
CTACACTAGAAGAACAGTATTTGGTATCTGCGCTCTGCTGAAGCCAGTTACCTTCGGAAAAAGAGTTGGTAGCTCTTGATCCGGCAAACAAACCACCGCTGGT
AGCGGTGGTTTTTTTGTTTGCAAGCAGCAGATTACGCGCAGAAAAAAAGGATCTCAAGAAGATCCTTTGATCTTTTCTACGGGGTCTGACGCTCAGTGGAACG
AAAACTCACGTTAAGGGATTTTGGTCATGAGATTATCAAAAAGGATCTTCACCTAGATCCTTTTAAATTAAAAATGAAGTTTTAAATCAATCTAAAGTATATA
TGAGTAAACTTGGTCTGACAGTTACCAATGCTTAATCAGTGAGGCACCTATCTCAGCGATCTGTCTATTTCGTTCATCCATAGTTGCCTGACTCCCCGTCGTG
TAGATAACTACGATACGGGAGGGCTTACCATCTGGCCCCAGTGCTGCAATGATACCGCGAGACCCACGCTCACCGGCTCCAGATTTATCAGCAATAAACCAGC
CAGCCGGAAGGGCCGAGCGCAGAAGTGGTCCTGCAACTTTATCCGCCTCCATCCAGTCTATTAATTGTTGCCGGGAAGCTAGAGTAAGTAGTTCGCCAGTTAA
TAGTTTGCGCAACGTTGTTGCCATTGCTACAGGCATCGTGGTGTCACGCTCGTCGTTTGGTATGGCTTCATTCAGCTCCGGTTCCCAACGATCAAGGCGAGTT
ACATGATCCCCCATGTTGTGCAAAAAAGCGGTTAGCTCCTTCGGTCCTCCGATCGTTGTCAGAAGTAAGTTGGCCGCAGTGTTATCACTCATGGTTATGGCAG
CACTGCATAATTCTCTTACTGTCATGCCATCCGTAAGATGCTTTTCTGTGACTGGTGAGTACTCAACCAAGTCATTCTGAGAATAGTGTATGCGGCGACCGAG
TTGCTCTTGCCCGGCGTCAATACGGGATAATACCGCGCCACATAGCAGAACTTTAAAAGTGCTCATCATTGGAAAACGTTCTTCGGGGCGAAAACTCTCAAGG
ATCTTACCGCTGTTGAGATCCAGTTCGATGTAACCCACTCGTGCACCCAACTGATCTTCAGCATCTTTTACTTTCACCAGCGTTTCTGGGTGAGCAAAAACAG
GAAGGCAAAATGCCGCAAAAAAGGGAATAAGGGCGACACGGAAATGTTGAATACTCATACTCTTCCTTTTTCAATATTATTGAAGCATTTATCAGGGTTATTG
TCTCATGAGCGGATACATATTTGAATGTATTTAGAAAAATAAACAAATAGGGGTTCCGCGCACATTTCCCCGAAAAGTGCCACCTG
